# MatBED_B&C: A 3-dimensional biologically effective dose analytic approach for the retrospective study of gamma knife radiosurgery in a B&C model

**DOI:** 10.1016/j.mex.2023.102320

**Published:** 2023-08-05

**Authors:** Ke Tang, Nan Zhang, Xiaodong Yuan, Zenghui Qian, Yang Li, Xu Feng

**Affiliations:** a Department of Neurosurgery, Chinese PLA General Hospital, 28 Fuxing Road, Beijing, PR China; b Department of Neurosurgery, Huashan Hospital, Fudan University, 12 Wulumuqi (Middle) Road, Shanghai, PR China; c Department of Radiology, The Eighth Medical Center of Chinese PLA General Hospital, 17 Heishanhu Road, Beijing, PR China; d Department of Neurosurgery, Beijing Tiantan Hospital, Capital Medical University, 119 Fanyang Road, Fengtai District, Beijing, PR China; e Department of Oral and Maxillofacial Surgery, Peking University School and Hospital of Stomatology, 22 Zhongguancun South Road, Beijing, PR China; f Department of Basic Medicine, Xiamen Medical College, 1999 Guankouzhong Road, Xiamen, Fujian Province, PR China

**Keywords:** Stereotactic, Three-dimensional model, Curve fitting, Dose distribution, Quality metrics, MatBED_B&C

## Abstract

The biological effect of irradiation is not solely determined by the physical dose. Gamma knife radiosurgery may be influenced by dose rate, beam-on-time, numbers of iso-centers, the gap between the individual iso-centers, and the dose‒response of various tissues. The biologically effective dose (BED) for radiosurgery considers these issues. Millions of patients treated with Models B and C provide a vast database to mine BED-related information. This research aims to develop MatBED_B&C, a 3-dimensional (3D) BED analytic approach, to generate a BED for individual voxels in the calculation matrix with related parameters extracted from Gammaplan. This approach calculates the distribution profiles of the BED in radiosurgical targets and organs at risk. A BED calculated on a voxel-by-voxel basis can be used to show the 3D morphology of the iso-BED surface and visualize the BED spatial distribution in the target. A 200 × 200 × 200 matrix can cover a greater range of the organ at risk. The BED calculated by MatBED_B&C can also be used to form BED-volume histograms to generate plan quality metrics, which will be studied in a retrospective study of gamma knife radiosurgery to guide future BED planning.

• We develop MatBED_B&C to calculate the 3D BED in radiosurgical targets.
• The BED of MatBED_B&C can visualize the BED spatial distribution profiles.
• The BED of MatBED_B&C will generate plan quality metrics studied in a retrospective study.

## Graphical abstract

**Figure:**
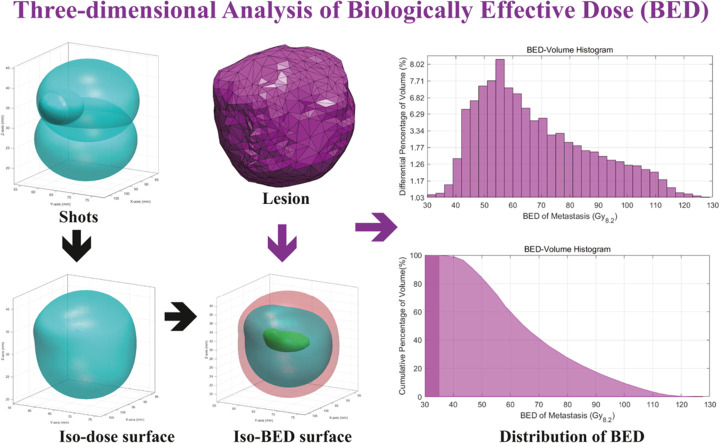

Specifications table

**Table:** 

Subject area:	Medicine and Dentistry
More specific subject area:	Stereotactic radiosurgery
Name of your method:	MatBED_B&C
Name and reference of original method:	Not applicable
Resource availability:	Six MATLAB program code files Dose_profile_4C_4 mm.m, Dose_profile_4C_8 mm.m, Dose_profile_4C_14 mm.m, Dose_profile_4C_18 mm.m, Total_dose.m, and BED.m are available on https://github.com/tangkeGitHubaccount/MatBED_B-C

## Method details

### Background information

The biological effect of irradiation results from the combined action of radiation damage and repair kinetics. The biologically effective dose (BED), developed as a radiotherapy concept, has been adopted in gamma knife radiosurgery (GKRS). The BED model considers the factors associated with the repair of sublethal damage related to radiotherapy. In GKRS, these factors include the collimator size, the number of iso-centers used, dose rate, beam-on-time, the gap between the individual iso-centers, and the dose‒response of tissues [1]. Consequently, the BED model for GKRS has been developed to consider the factors expressed as the following equations [2,3]:

(1) $$BED=D_{T}+\frac{1}{\alpha /\beta }\left[\frac{\Phi \left(\Xi ,\;\mu_{1}\right)+c\Phi \left(\Xi ,\;\mu_{2}\right)}{1+c}\right]\sum_{i=1}^{N}d_{i}^{2}$$

where Φ(Ξ,μ) is expressed as:

(2) $$\Phi \left(\Xi ,\;\mu \right)=\frac{\frac{2}{\mu }\sum_{j=1}^{N}\left[d_{j}^{2}\frac{\left\{\delta t_{j}-\frac{1}{\mu }\left(1-e^{-\mu \delta t_{j}}\right)\right\}}{\delta t_{j}^{2}}-\frac{1}{\mu }\sum_{i=1}^{i=j-1}d_{i}d_{j}\frac{e^{-\mu \left(t_{j}-t_{i}\right)}\left(e^{\mu \delta t_{i}}-1\right)\left(e^{-\mu \delta t_{j}}-1\right)}{\delta t_{i}\delta t_{j}}\right]}{\sum_{k=1}^{N}d_{k}^{2}}$$

D_T_ is the total physical dose. The α/β ratio reflects the dose response of various tissues. The equation combines the fast and slow components of repair (µ_1_ and µ_2_) in a partition model (partition coefficient c). *d_i_, d_j_*, and *d_k_* are the dose distributions of the *i*th, *j*th, and *k*th shots, and *δt_i_* and *δt_j_* are the irradiation durations of the *i*th and *j*th shots, respectively. *t_i_* and *t_j_* are the initiation times of the *i*th and jth shots, respectively. Thus, the value of *t_j_*-*t_i_* is equal to *δt_i_* +*δt_gap_. δt_gap_* is the duration of the gap. Here, N is the number of iso-centers used. Eqs. (1) and (2) can be used to generate a BED for individual voxels. To reduce the calculation load, two approaches can be used. One method is extracting the physical dose distribution directly from the availability of the GammaPlan, as introduced by Hopewell et al. [1]. This strategy, as previously mentioned, is GammaPlan version dependant. However, when the availability of the research version of GammaPlan is lacking, significant computational load may make the BED calculation in each voxel problematic. The BED is calculated based on the original physical dose distribution on a voxel-by-voxel basis. Indeed, physical dose distribution calculation on the MR or CT images slice by slice formed a significant computational load. Accordingly, Jones et al. summarized simplified approaches for BED calculation based on higher and lower total treatment times [4]. Graffeo et al.'s study used a monoexponential fit equation to generate an estimated BED from treatment time and margin dose pairs [5]. These simplified approaches have the limitation of more systematic error than an actual BED calculated on a voxel-by-voxel basis. The report of Klinge et al. used a 31 × 31 × 31 matrix covering the selected region to calculate the BED for each voxel. However, only the BED on the prescription dose iso-surface was calculated [6]. Thus, the calculated BED did not represent a comprehensive BED distribution in the target. In addition, to calculate the BED distribution in the organ at risk, such as a pyramidal tract with a length of more than 150 mm, the 31 × 31 × 31 matrix may hardly cover the range. Therefore, comprehensive evaluations of the BED spatial distribution profiles still face challenges. Another calculation approach to reduce the calculation load is calculating the physical dose distribution using three-dimensional (3D) coordinate values, dose rate, and beam-on time of each shot extracted from the GammaPlan. However, this is version-independent. The latter approach calculates the dose falloff based on the maximum central radiation doses of each shot rather than directly extracting the physical dose distribution. In our study, we used the latter strategy.

Since a GKRS treatment plan devised by different physicians has endless permutations regarding the location and number of different iso-centers, even with the same prescription dose, using physical dose distribution to evaluate the quality of a treatment plan is insufficient. Consequently, BED combined the above-mentioned indicators into a single compound indicator in seeking to predict the treatment outcome. For high-dose, single-fraction treatments, for example, Smith et al. has reported the dose rate effects following Gamma Knife surgery for vestibular schwannomas [7]. Further, Villafuerte et al.'s report has shown the correlation between BED and local control after radiosurgery for acoustic neuromas [8]. Two studies of Tuleasca et al. also showed that BED was associated with linear tumour volume changes and hearing preservation after stereotactic radiosurgery for vestibular schwannomas [9,10]. Another study of Tuleasca et al. showed the correlation between GKRS BED and obliteration of unruptured arteriovenous malformation [11]. In addition, as mentioned above, Graffeo et al.'s studies used estimated BED as the variable to predict hypopituitarism after single-fraction pituitary adenoma radiosurgery and predict outcomes for acromegaly [5,12]. One major limitation of these studies is that the estimated BED did not consider the location (3D coordinate values) of different iso-centers. Notably, the iso-center location in the GKRS target is an important factor influencing the plan quality. Thus, we developed a 3D BED analytic approach to incorporate the 3D coordinate values of each GKRS iso-center into a BED computational model. It is also worth noting that the 3D BED model in our study is still a model just based on the hypothesis that BED or dose rate is a factor for SRS treatments. To refine the BED model and explore the guiding value of the model for GKRS, the first step is to use the model in the retrospective study and validate the hypothesis. This is our motivation for developing MatBED_B&C, a 3D BED analytic approach.

Gamma knife Models B and C are representative models in the era of stereotactic radiosurgery. In the context of the development of BED analysis methods, millions of brain disorder patients treated worldwide with Models B and C provide a vast database to mine BED-related information. Thus, it is essential to develop a BED analytic approach for the retrospective study of GKRS in the B&C model. MatBED_B&C is a 3-dimensional (3D) BED analytic approach to generate a BED for individual voxels. The parameters involving 3D coordinate values, dose rate, and beam-on time of each shot were extracted from the Gammaplan of the B&C model to generate our BED calculation matrix. These parameters were the output values of the GammaPlan with related versions of Models B and C. Thus, the software version had no significant impact on our calculation matrix. The MATLAB R2020a editor (http://www.mathworks.com/products/matlab/) developed the 3D BED analytic approach for the GammaPlan of the B&C model. The workflow of BED analysis contains two main steps: (1) for the solver of the 3D dose profiles for the ellipsoid expansion coefficient, we deploy the Gaussian function to fit the dose profiles. The data for dose profiles can be acquired in TMR10 [13], [14], [15] or by using a radiochromic film dosimeter in the phantom [16], in which the latter forms a curve in the dose profiles for individualized GKRS instruments. (2) A 3D BED calculation is deployed based on spatial distribution profiles of the physical dose. The essential step is to use the 3D coordinate values of each iso-center to calculate the dose distribution and BED distribution in a 200×200×200 matrix. For the B&C model, an iso-center is generated by a 4, 8, 14, or 18 mm collimator. Each collimator has an association between dose falloff and 3D coordinate values.

### Solver of the 3D dose profiles for the ellipsoid expansion coefficient

**Fig. 1:**
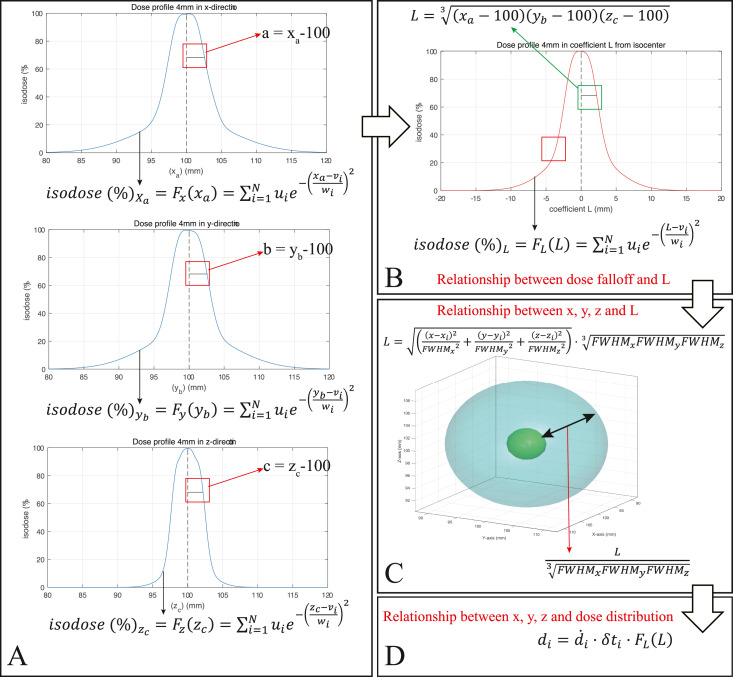
A diagram of the computational pipeline. A: Examples show that the lengths of the semimajor axis, indicated by a, b, and c with horizontal solid black lines in a red square frame, in the x-direction, y-direction, and z-direction correspond to the 70% iso-dose in the dose profiles of an iso-center at coordinates (100, 100, 100). Next, x_a_, y_b_, and z_c_ are the abscissa values at the dose percentage in the x-direction, y-direction, and z-direction, respectively. The curves in the dose profiles in the x-direction, y-direction, and z-direction are used to fit the dose falloff by a Gaussian function (blue curves indicated by black arrows). The dose falloff profiles show that the dose decays by its percentage on either side of the reference point (100% iso-dose). The a, b, and c are the abscissa differences between the reference point and any point of the dose decay curve in the x-direction, y-direction, and z-direction, respectively. Here, a, b, and c can take negative values due to the purpose of fitting the two-sided dose falloff. The percent dose at the point of the dose decay curve can be any percentage, for example, 90%, 70%, or 50% et al. Here we selected 70% as an example. B: L (indicated by horizontal solid black line in a green square frame) is the geometric mean of a, b, and c. L can take negative values to express dose falloff in another direction (with a portion indicated by a red square frame). The curve is also used to fit the dose falloff by a Gaussian function (red curves indicated by black arrows). *N, u_i_, v_i_,* and *w_i_* take respective values corresponding to x_a_, y_b_, z_c_, and L, respectively. C: An expanding ellipsoidal is used to represent the relationship between 3D coordinate values (x, y, z) and L. The expansion and contraction of the ellipsoidal (indicated by a black two-direction arrow) is controlled by $\frac{L}{\sqrt[{3}]{FWHM_{x}FWHM_{y}FWHM_{z}}}$ (indicated by a red arrow). D: The relationship between dose falloff and 3D coordinate values (x, y, z) is used to calculate the physical dose distribution. 3D: 3-dimensional.

Fig. 1 shows a diagram of the computational pipeline. First, we set the coefficient L of ellipsoid expansion to form an association between dose falloff and 3D coordinate values. Then, for simplicity, the iso-dose contours are calculated in an expanding ellipsoidal around the iso-center. As a result, the ellipsoid equation can be written as follows:

(3) $$\frac{{\left(x-x_{i}\right)}^{2}}{a^{2}}+\frac{{\left(y-y_{i}\right)}^{2}}{b^{2}}+\frac{{\left(z-z_{i}\right)}^{2}}{c^{2}}=1$$

*(x, y, z)* and *(x_i_, y_i_, z_i_)* are the 3D coordinate values of one dose-distribution point and iso-center for the *i*th irradiation, respectively. a, b, and c are the lengths of the semimajor axis in the x-direction, y-direction, and z-direction, respectively. A coefficient L of ellipsoid expansion is set as:

(4) $$a^{2}=\frac{L^{2}\cdot FWHM_{x}^{2}}{\sqrt[{3}]{FWHM_{x}^{2}\cdot FWHM_{y}^{2}\cdot FWHM_{z}^{2}}}={\left(x_{a}-100\right)}^{2}$$

(5) $$b^{2}=\frac{L^{2}\cdot FWHM_{y}^{2}}{\sqrt[{3}]{FWHM_{x}^{2}\cdot FWHM_{y}^{2}\cdot FWHM_{z}^{2}}}={\left(y_{b}-100\right)}^{2}$$

(6) $$c^{2}=\frac{L^{2}\cdot FWHM_{z}^{2}}{\sqrt[{3}]{FWHM_{x}^{2}\cdot FWHM_{y}^{2}\cdot FWHM_{z}^{2}}}={\left(z_{c}-100\right)}^{2}$$

The x_a,_ y_b_, and z_c_ are the abscissa values at the dose percentage in the x-direction, y-direction, and z-direction, respectively. In the dose profiles of an iso-center at the Leksell® coordinates (100, 100, 100) described in the TMR10 [13], [14], [15], we can also use a radiochromic film dosimeter in the phantom to acquire data, forming a curve in the dose profiles for individualized GKRS instruments [16]. The reference point with coordinates *(100,100,100)* is the position of the central maximum dose. The coefficient of ellipsoid expansion, L, positively correlates with the distance between a point *(x_a_, y_b_, z_c_)* on an ellipsoid and the reference point *(100,100,100)*. The ellipsoid expansion starts from the reference point and extends to cover the whole 3D space. The distance component in the x-direction, y-direction, and z-direction is *(x_a_-100), (y_b_-100)*, and *(z_c_-100*), respectively. According to the ellipsoid equation Eq. (3)), *a, b*, and *c* are the lengths of the semimajor axis in the x-direction, y-direction, and z-direction, respectively. Consequently, we get: $a=|x_{a}-100|$. Thus, in Eq. (4), $a^{2}={\left(x_{a}-100\right)}^{2}$. Similarly, in Eq. (5), $b^{2}={\left(y_{b}-100\right)}^{2}$, and in Eq. (6), $c^{2}={\left(z_{c}-100\right)}^{2}$. In the dose profiles, *FWHM* is the full width at half maximum dose percentage values of one shot caused by the 4, 8, 14, and 18 mm collimators. *FWHM_x_, FWHM_y_*, and *FWHM_z_* are the *FWHM* values in the x-direction, y-direction, and z-direction, respectively. Next, we use three relations: $L=\;\frac{a\cdot \sqrt[{3}]{FWHM_{x}\cdot FWHM_{y}\cdot FWHM_{z}}}{FWHM_{x}}$, $L=\frac{b\cdot \sqrt[{3}]{FWHM_{x}\cdot FWHM_{y}\cdot FWHM_{z}}}{FWHM_{y}}$, and $L=\frac{c\cdot \sqrt[{3}]{FWHM_{x}\cdot FWHM_{y}\cdot FWHM_{z}}}{FWHM_{z}}$, respectively. Consequently, in Eqs. (4)–((6), $a^{2}=\frac{L^{2}\cdot FWHM_{x}^{2}}{\sqrt[{3}]{FWHM_{x}^{2}\cdot FWHM_{y}^{2}\cdot FWHM_{z}^{2}}}$, $b^{2}=\frac{L^{2}\cdot FWHM_{y}^{2}}{\sqrt[{3}]{FWHM_{x}^{2}\cdot FWHM_{y}^{2}\cdot FWHM_{z}^{2}}}$, and $c^{2}=\frac{L^{2}\cdot FWHM_{z}^{2}}{\sqrt[{3}]{FWHM_{x}^{2}\cdot FWHM_{y}^{2}\cdot FWHM_{z}^{2}}}$. Since the ellipsoid expansion can cover any point *(x, y, z)* of the whole 3D space, meanwhile, the reference point can be set as *(x_i_, y_i_, z_i_)*, substituting Eqs. (4)–(6) into Eq. (3), we achieve the following:

(7) $$L=\sqrt{\left(\frac{{\left(x-x_{i}\right)}^{2}}{FWHM_{x}^{2}}+\frac{{\left(y-y_{i}\right)}^{2}}{FWHM_{y}^{2}}+\frac{{\left(z-z_{i}\right)}^{2}}{FWHM_{z}^{2}}\right)}\cdot \sqrt[{3}]{FWHM_{x}FWHM_{y}FWHM_{z}}$$

On the one hand, to express the dose profiles of collimators, the relationship between dose falloff and L can be fitted by the Gaussian function of the MATLAB Curve Fitting Tool.

First, the ellipsoid volume can be calculated as follows:

(8) $$V_{ellipsoidal}=\frac{4}{3}\pi abc$$

Substituting Eqs. (4)–(6) into Eq. (8), when Eqs. (4)–(6) are multiplied, the FWHMx, FWHMy, and FWHMz elimination are completed. Thus, we achieve the following:

(9) $$L=\sqrt[{3}]{\left(x_{a}-100\right)\left(y_{b}-100\right)\left(z_{c}-100\right)}$$

where L becomes the geometric mean of the lengths of the semimajor axis in the x-direction, y-direction, and z-direction. As Eq. (8) shows, the geometric meaning (L) can indicate the radius of a sphere with an equal volume of ellipsoid expressed by Eq. (1).

However, as Fig. 1A and B show, a, b, c, and L can take negative values due to the purpose of fitting the two-sided dose falloff. Since the dose falloff is on two sides, not one side in the x, y, and z directions, the negative values of a, b, c, and L here express dose falloff in another direction.

Next, we adopt the Gaussian function of the MATLAB Curve Fitting Tool to fit the dose profiles. The curve in the dose profiles is used to achieve the following relations:

(10) $$isodose\;{\left(\%\right)}_{X_{a}}=F_{x}\left(x_{a}\right)=\sum_{i=1}^{N}u_{i}e^{-{\left(\frac{x_{a}-v_{i}}{w_{i}}\right)}^{2}}$$

(11) $$isodose\;{\left(\%\right)}_{y_{b}}=F_{y}\left(y_{b}\right)=\sum_{i=1}^{N}u_{i}e^{-{\left(\frac{y_{b}-v_{i}}{w_{i}}\right)}^{2}}$$

(12) $$isodose\;{\left(\%\right)}_{z_{c}}=F_{z}\left(z_{c}\right)=\sum_{i=1}^{N}u_{i}e^{-{\left(\frac{z_{c}-v_{i}}{w_{i}}\right)}^{2}}$$

Then, we substitute Eqs. (10)–(12) into Eq. (9) to achieve the following relation:

(13) $$isodose\;{\left(\%\right)}_{L}=F_{L}\left(L\right)=\sum_{i=1}^{N}u_{i}e^{-{\left(\frac{L-v_{i}}{w_{i}}\right)}^{2}}$$

Here, N is the number of terms (Gaussian function fitting dose falloff). Eqs. (10)–(13) express the corresponding dose profile for each collimator. During the curve fitting, parameters involving *N, u_i_, v_i_,* and *w_i_* assume different values corresponding to Eqs. (10)–(13) to fit the respective dose falloff. We thus resolve the dose profiles of the coefficient L for all collimators. *u_i_, v_i_*, and *w_i_* are fit coefficients of the Gaussian function. The details of the curve fitting are provided in Supplementary Material A.

On the other hand, the relationship between 3D coordinate values (x, y, z) and L is formed by Eq. (7), which is achieved by substituting Eqs. (4)–(6) into Eq. (3). Here, the implication of L is a correlation coefficient of gradually expanding an ellipsoid with 3D coordinate values as parameters (Fig. 1C). According to Eq. (7), we obtain:

$\frac{L}{\sqrt[{3}]{FWHM_{x}FWHM_{y}FWHM_{z}}}=\sqrt{\left(\frac{{\left(x-x_{i}\right)}^{2}}{FWHM_{x}^{2}}+\frac{{\left(y-y_{i}\right)}^{2}}{FWHM_{y}^{2}}+\frac{{\left(z-z_{i}\right)}^{2}}{FWHM_{z}^{2}}\right)}$, where $\frac{L}{\sqrt[{3}]{FWHM_{x}FWHM_{y}FWHM_{z}}}$can be used to control the expansion of the ellipsoid (the length changes of semimajor axes). When $\frac{L}{\sqrt[{3}]{FWHM_{x}FWHM_{y}FWHM_{z}}}=1/2\;\left(also\;means:\;L=\sqrt[{3}]{\left(FWHM_{x}/2\right)\left(FWHM_{y}/2\right)\left(FWHM_{z}/2\right)}\right)$, we can obtain $\frac{{\left(x-x_{i}\right)}^{2}}{{\left(FWHM_{x}/2\right)}^{2}}+\frac{{\left(y-y_{i}\right)}^{2}}{{\left(FWHM_{y}/2\right)}^{2}}+\frac{{\left(z-z_{i}\right)}^{2}}{{\left(FWHM_{z}/2\right)}^{2}}=1$, which can form an ellipsoid indicating the 3D shape of a collimator shot with the corresponding *FWHM*.

Consequently, based on the relationships between dose falloff and L and between 3D coordinate values (x, y, z) and L, we obtain the relationship between dose falloff and 3D coordinate values (x, y, z). Finally, we calculate the physical dose distribution using the 3D coordinate values, dose rate, and beam-on time of each shot as follows.

### BED calculation based on spatial distribution profiles of the physical dose

Then, the dose distribution is calculated as follows:

(14) $$d_{i}={\dot{d}}_{i}\cdot \delta t_{i}\cdot F_{L}\left(L\right)$$

**Fig. 2:**
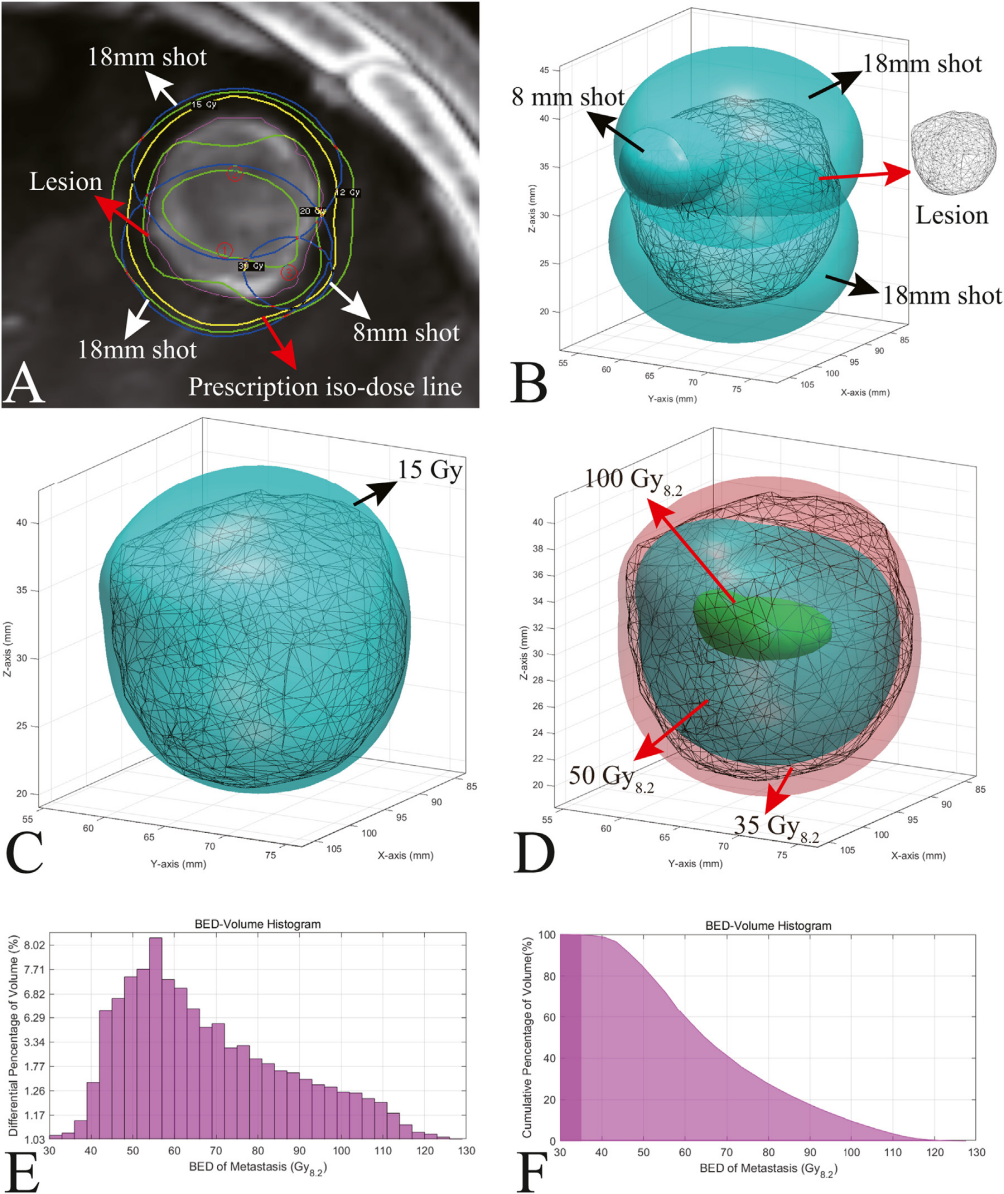
A representative example of a 3D BED analytic approach. A: Target of a case with gamma knife radiosurgery. Layouts of shots (indicated by a blue circle and white arrow) in a lesion (magenta contour indicated by red arrow). A yellow contour and red arrow indicate the prescription iso-dose line. B: The example shows shot contours (indicated by black arrows) for 8 mm and 18 mm collimators. The spatial relationship between the shots and the irradiated object (indicated by red arrows) was visualized. C: 3D iso-surface (cyan contour indicated by black arrow) of 15 Gy enclosing the lesion. The contour of the iso-surface represents 15 Gy at the 40% iso-surface. D: Distribution of 3D iso-BED shows iso-surfaces of 35 Gy_8.2_ (red contour), 50 Gy_8.2_ (cyan contour), and 100 Gy_8.2_ (green contour). The contours of the three iso-surfaces represent the 3D distributions of the BED. E: Differential BED-volume histograms for the example. F: Cumulative BED-volume histograms for the example. BED: biologically effective dose, 3D: 3-dimensional.

*d_i_*, ${\dot{d}}_{i}$, and *δt_i_* are the dose distribution, iso-center dose rate, and irradiation duration of the *i*th shot, respectively. A contour of one shot is expressed as an iso-dose of half the maximum dose. Thus, we visualize the spatial morphology and position of a shot using the 3D coordinate values of each iso-center of the B&C model during a retrospective study. If we input a 3D model of a radiosurgical target Fig. 2A), we achieve the spatial relationship between the shot and the irradiated objects (Fig. 2B). Then, the iso-dose surface visualizes the total dose of all iso-centers (Fig. 2C). Accordingly, we calculate a BED for individual voxels based on physical dose using Eqs. (1) and ((2) and visualize the spatial distribution of the BED in the radiosurgical target (Fig. 2D). Since the calculation grid size of the 200 × 200 × 200 matrix is 1 mm, the volume is calculated by summing the volume of voxels for the target. According to the 3D coordinate value of each voxel of the target, we generate BED-volume histograms according to the 3D BED distributions (Fig. 2E and F). The visualization details for the shot, iso-dose surface, iso-BED surface, and formation of the BED-volume histogram are provided in Supplementary Material B. The BED-volume histograms can be used to obtain plan quality metrics such as percentage volume BED, iso-BED volume, conformity index, and gradient index. These BED metrics will be studied in a retrospective study of gamma knife radiosurgery to guide future BED planning.

**Fig. 3:**
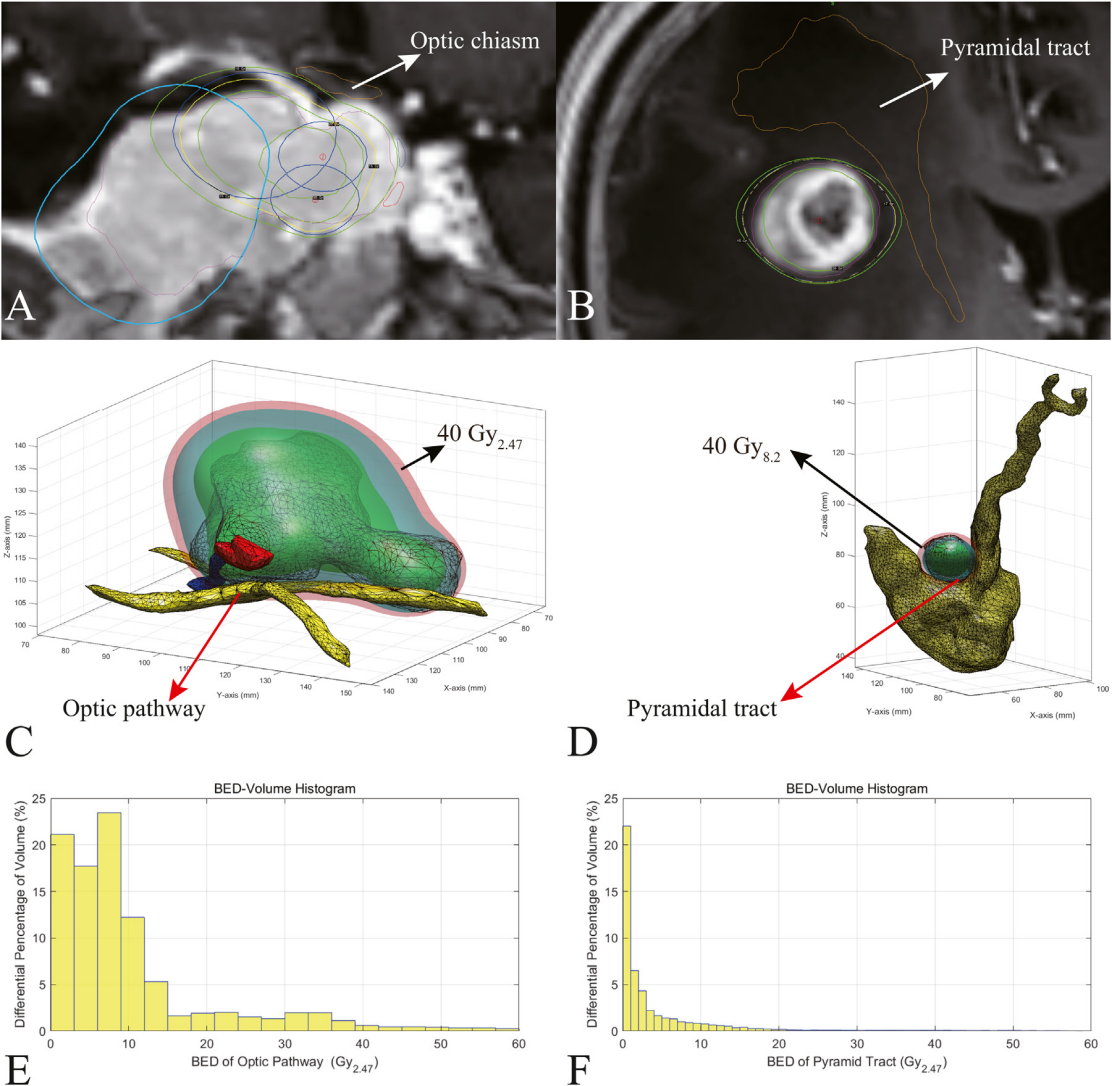
Examples of a 3D BED analysis for organs at risk. A: A case with optic chiasm (indicated by yellow contour with white arrow) adjacent to a parasellar target. B: A case with a pyramidal tract (indicated by yellow contour with white arrow) adjacent to a target in the frontal lobe. C: 3D view shows the spatial relationship between the optic pathway (indicated by yellow contour with red arrow) and the BED iso-surface enclosing the lesion. The BED iso-surfaces of 40 Gy_2.47_ are indicated by red transparent contours with black arrows. D: 3D view shows the spatial relationship between the pyramidal tract (indicated by yellow contour with red arrow) and the BED iso-surface enclosing the lesion. The BED iso-surfaces of 40 Gy_8.2_ are indicated by red transparent contours with black arrows. E: Differential BED-volume histograms for the optic pathway. F: Differential BED-volume histograms for the pyramidal tract. BED: biologically effective dose, 3D: 3-dimensional.

When required to evaluate BED distribution in the organs at risk, such as the optic pathway (Fig. 3A) or pyramidal tract (Fig. 3B) adjacent to the radiosurgical targets, MatBED_B&C can be used to show the spatial relationship between the BED iso-surface and the organs at risk (Fig. 3C and D). The spatial range of the organ at risk may be much greater than that of the surgical target. The 200 × 200 × 200 matrix can cover the range of organs at risk. Accordingly, a BED-volume histogram scan was also generated (Fig. 3E and F). The BED-volume histograms can be used to obtain plan quality metrics such as percentage volume BED, iso-BED volume, conformity index, and gradient index. These BED metrics will be studied in a retrospective study of gamma knife radiosurgery to guide future BED planning. Since the Perfexion/Icon models have the 4, 8, and 16 mm collimators, each of which has 8 sectors. Different combinations of the sectors make the calculation approach of dose distribution more challenging. Further work on the analytic method for Perfexion/Icon models is pending. This study's 4C model analytical approach provides a foundation for future studies.

### Limitations and further works in this direction

In the modern GK models (Perfexion, Icon, and Esprit), the radiation units consist of a fixed conical collimation system and 192 ^60^Co sources equally distributed over 8 sectors in a cylindrical configuration. The three available collimation channels, labelled as 4, 8, and 16 mm, allow the use of composite shot [17]. It means there are numerous combinations of different sectors with different collimation channels. Therefore, the dose falloff profiles of each shot of the modern GK models are more obviously more complex than those of the B or C GK models. In contrast, the method of MatBED_B&C can directly calculate the dose falloff profiles of one shot caused by the 4, 8, 14, and 18 mm collimators of the B or C GK models without beam channel blocking. Under no channel blocking condition, the dose falloff profiles of each shot in the x-direction, y-direction, and z-direction approximately follow a normal distribution. Consequently, the Gaussian function can fit the dose profiles for MatBED_B&C. One limitation of MatBED_B&C is that it cannot calculate the dose profiles of shots with channel blocking. The computational pipeline of MatBED_B&C is also applicable to calculate the dose falloff profile of one collimator for Perfexion and Icon only if all 8 sectors open without using a composite shot. The current method does not apply to the composite shot of Perfexion and ICON with or without beam channel blocking. To solve this problem, further work is being carried out to calculate the dose falloff of each sector rather than each shot for Perfexion and ICON.

## Conclusions

We developed MatBED_B&C, a GKRS 3D BED analytic approach for retrospective study with a reduced computational burden to generate a BED for individual voxels. The BED calculation is based on spatial distribution profiles of the physical dose calculated from the 3D coordinate values of each iso-center. The BED of MatBED_B&C can visualize the spatial relationship between the BED iso-surface and radiosurgical target or organs at risk and generate a BED-volume histogram.

## Ethics statements

The institutional ethics committee approved the study and exempted informed consent.

## CRediT authorship contribution statement

**Ke Tang:** Conceptualization, Software, Resources, Formal analysis, Supervision, Writing – original draft, Writing – review & editing. **Nan Zhang:** Data curation, Supervision, Formal analysis, Writing – review & editing. **Xiaodong Yuan:** Conceptualization, Writing – review & editing, Formal analysis, Supervision, Resources, Supervision. **Zenghui Qian:** Data curation, Formal analysis, Writing – review & editing. **Yang Li:** Data curation, Visualization, Formal analysis, Writing – review & editing. **Xu Feng:** Data curation, Formal analysis, Writing – review & editing.

## Declaration of Competing Interest

The authors declare that they have no known competing financial interests or personal relationships that could have appeared to influence the work reported in this paper.

## Data Availability

I have shared the link to my data/code at the Attach File step.

## Appendix Supplementary materials

Image, application 1

Image, application 2
