# Supplementary material for: MatBED_B&C: A 3-dimensional biologically effective dose analytic approach for the retrospective study of gamma knife radiosurgery in a B&C model
Source: MethodsX. 2023 Aug 5;11:102320. doi: 10.1016/j.mex.2023.102320 (PMC10433126; doi:10.1016/j.mex.2023.102320)
Supplement: Supplementary file 1 [file mmc1.docx]

***Supplementary Material A***

**Fitting the curve in the dose profiles of TMR 10[1-3] for the** **4 mm, 8 mm, 14 mm, and 18 mm collimator**

The relations between isodose (%) and x_a_, y_b_, z_c_, and L was achieved by MATLAB Curve Fitting Tool.

**Supplemental Figure A1**

**The coefficients of** **the 4 mm collimator in the x direction**


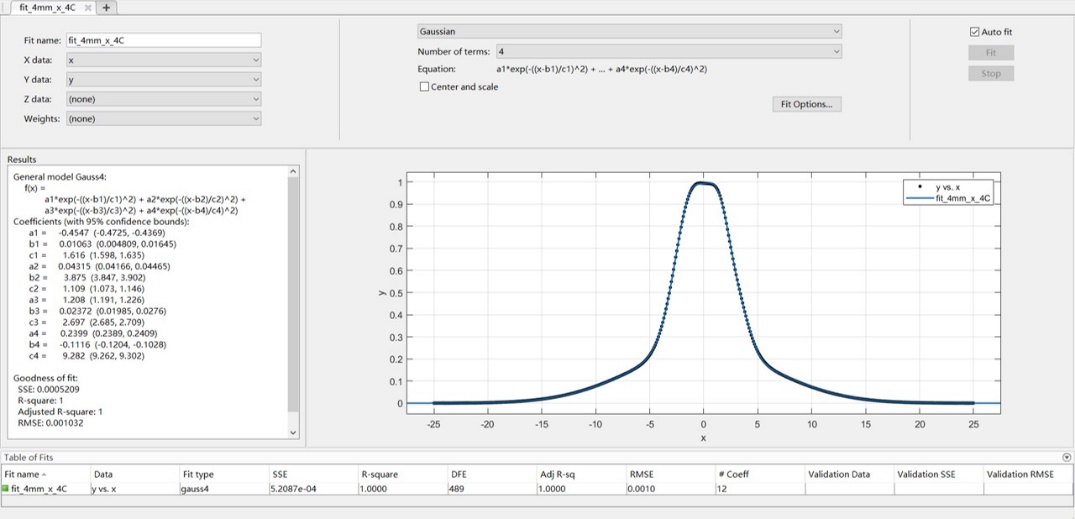


The curve in the dose profiles of the 4 mm collimator in the x direction was used to achieve the coefficients of the Gaussian function. Dose profiles of the Gaussian Fit model are indicated by a cyan curve line, and actual dose profiles are shown by a black dot and curve line. The steps following were the same as described here. With respect to $F_{x}\left( x_{a} \right)=\sum_{i=1}^{N} u_{i}e^{-\left( \frac{x_{a}-v_{i}}{w_{i}} \right)^{2}}$, here, a1-3 in MATLAB Curve Fitting Tool indicates *u_i_*. b1-3 indicates *v_i_*, and c1-3 indicates *w_i_*. N is equal to 3 for this case.

**Supplemental Figure A2**

**The relations between isodose (%) and x_a_ of the 4 mm collimator**


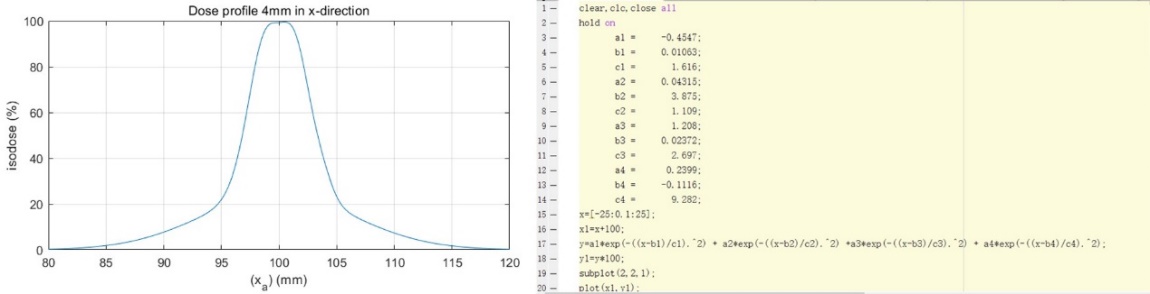


The relations between isodose (%) and x_a_ of the 4 mm collimator was calculated by the fitting coefficient.

**Supplemental Figure A3**

**The coefficients of the 4 mm collimator in the y direction**


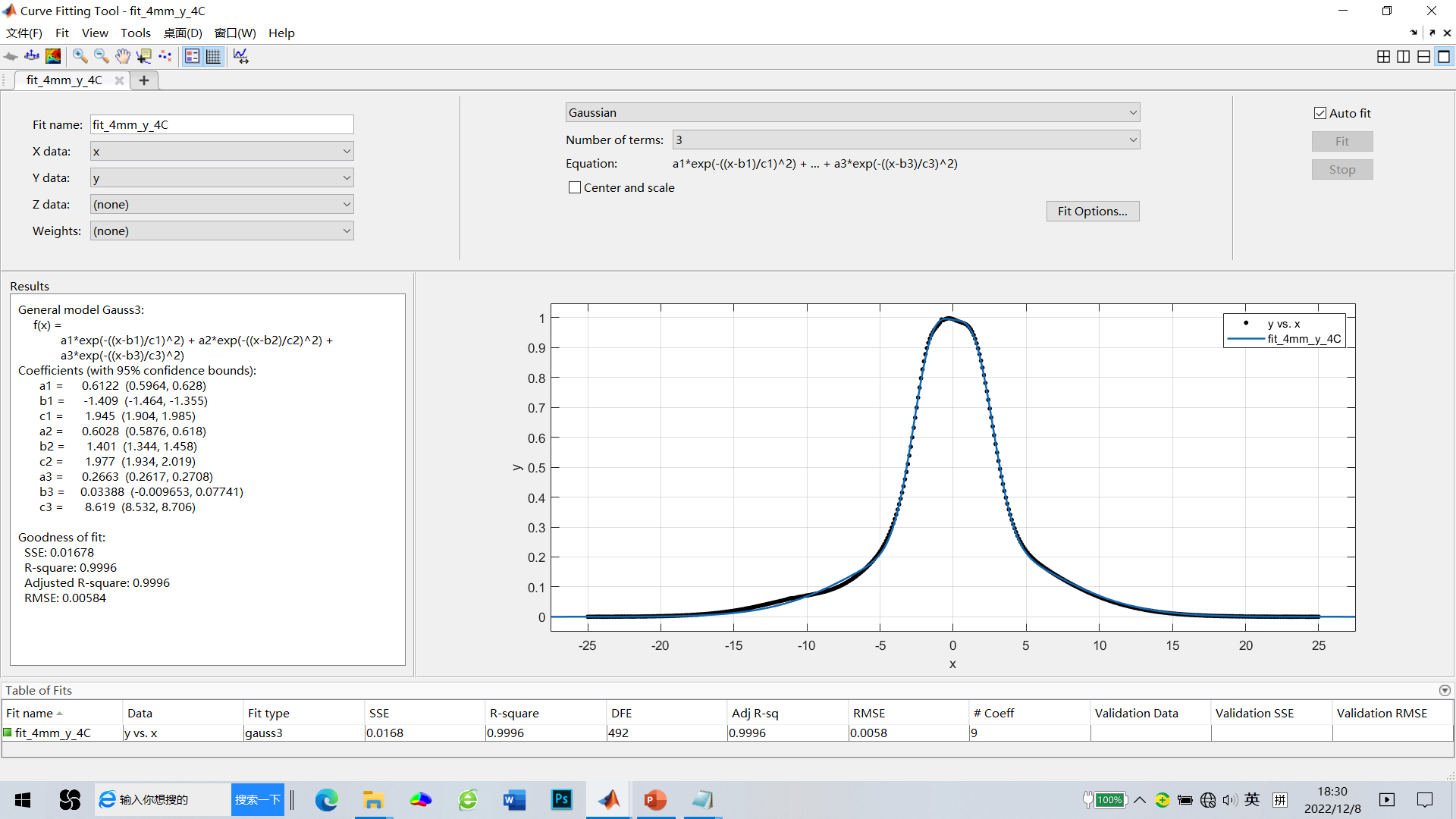


The curve in the dose profiles of the 4 mm collimator in the y direction was used to achieve the coefficients of the Gaussian function. With respect to $F_{y}\left( y_{b} \right)=\sum_{i=1}^{N} u_{i}e^{-\left( \frac{y_{b}-v_{i}}{w_{i}} \right)^{2}}$, here, a1-3 in MATLAB Curve Fitting Tool indicates *u_i_*. b1-3 indicates *v_i_*, and c1-3 indicates *w_i_*, N is equal to 3 for this case.

**Supplemental Figure A4**

**The relations between isodose (%) and y_b_ of the 4 mm collimator**


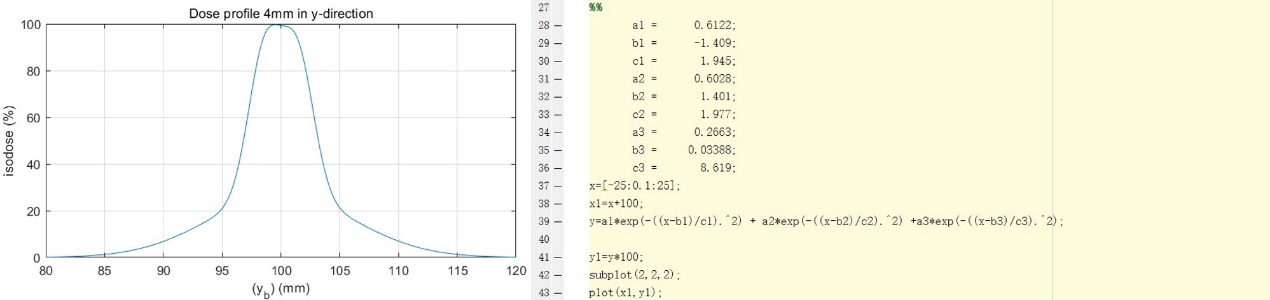


The relations between isodose (%) and y_b_ of the 4 mm collimator was calculated by the fitting coefficient.

**Supplemental Figure A5**

**The coefficients of the 4 mm collimator in the z direction**


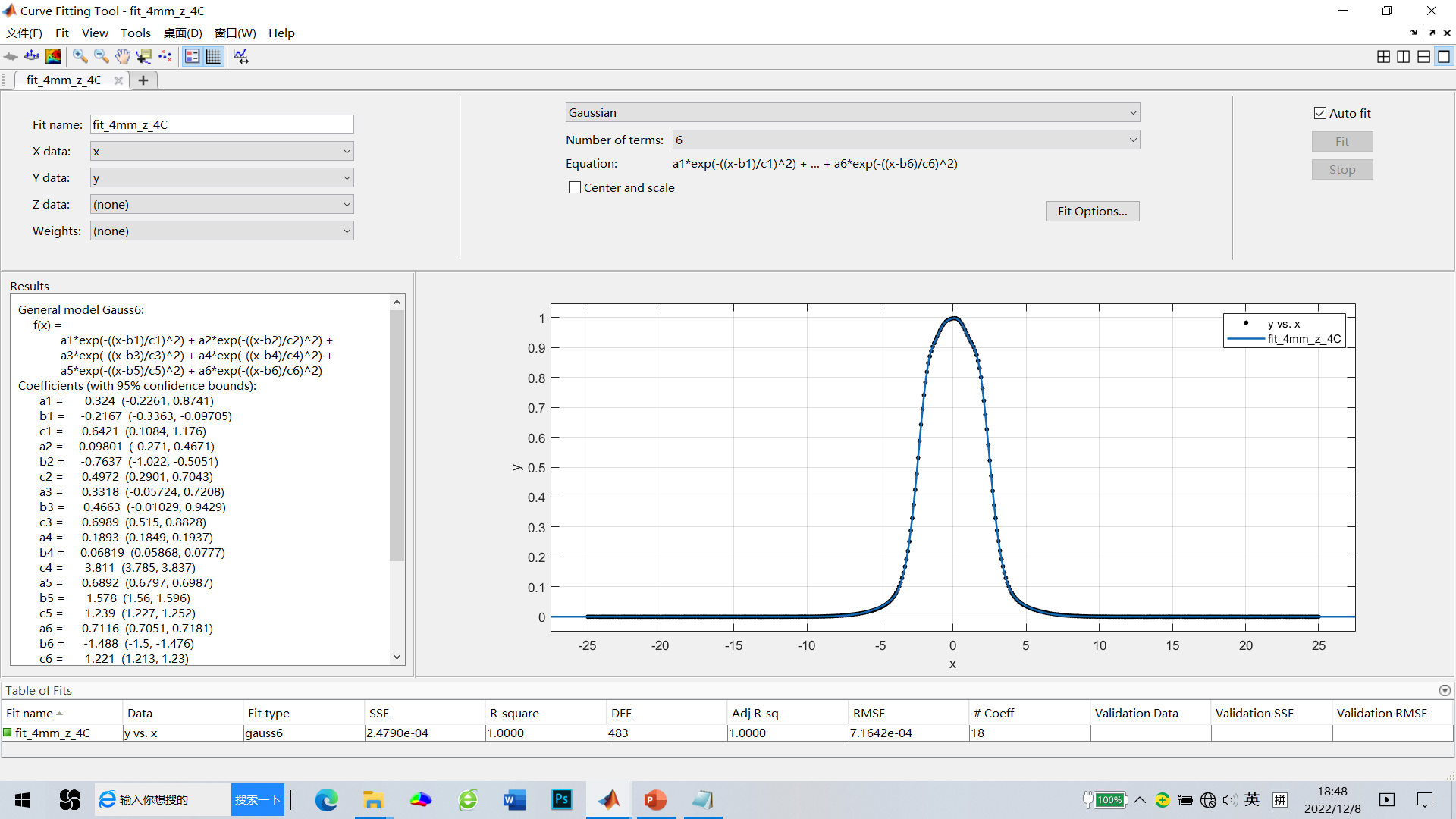


The curve in the dose profiles of the 4 mm collimator in the z direction was used to achieve the coefficients of the Gaussian function. With respect to $F_{z}\left( z_{c} \right)=\sum_{i=1}^{N} u_{i}e^{-\left( \frac{z_{c}-v_{i}}{w_{i}} \right)^{2}}$, here, a1-6 in MATLAB Curve Fitting Tool indicates *u_i_*. b1-6 indicates *v_i_*, and c1-6 indicates *w_i_*, N is equal to 6 for this case.

**Supplemental Figure A6**

**The relations between isodose (%) and z_c_ of the 4 mm collimator**


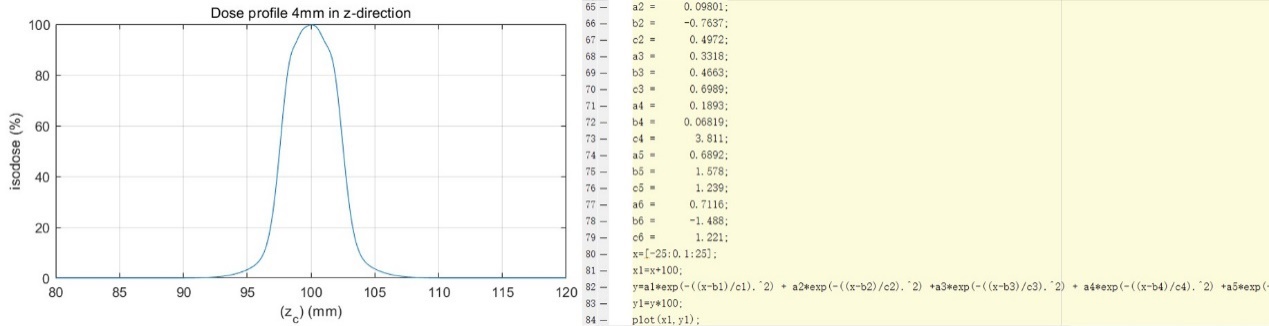


The relations between isodose (%) and z_c_ of the 4 mm collimator was calculated by the fitting coefficient.

**Supplemental Figure A7**

**The coefficients of the 4 mm collimator for L**


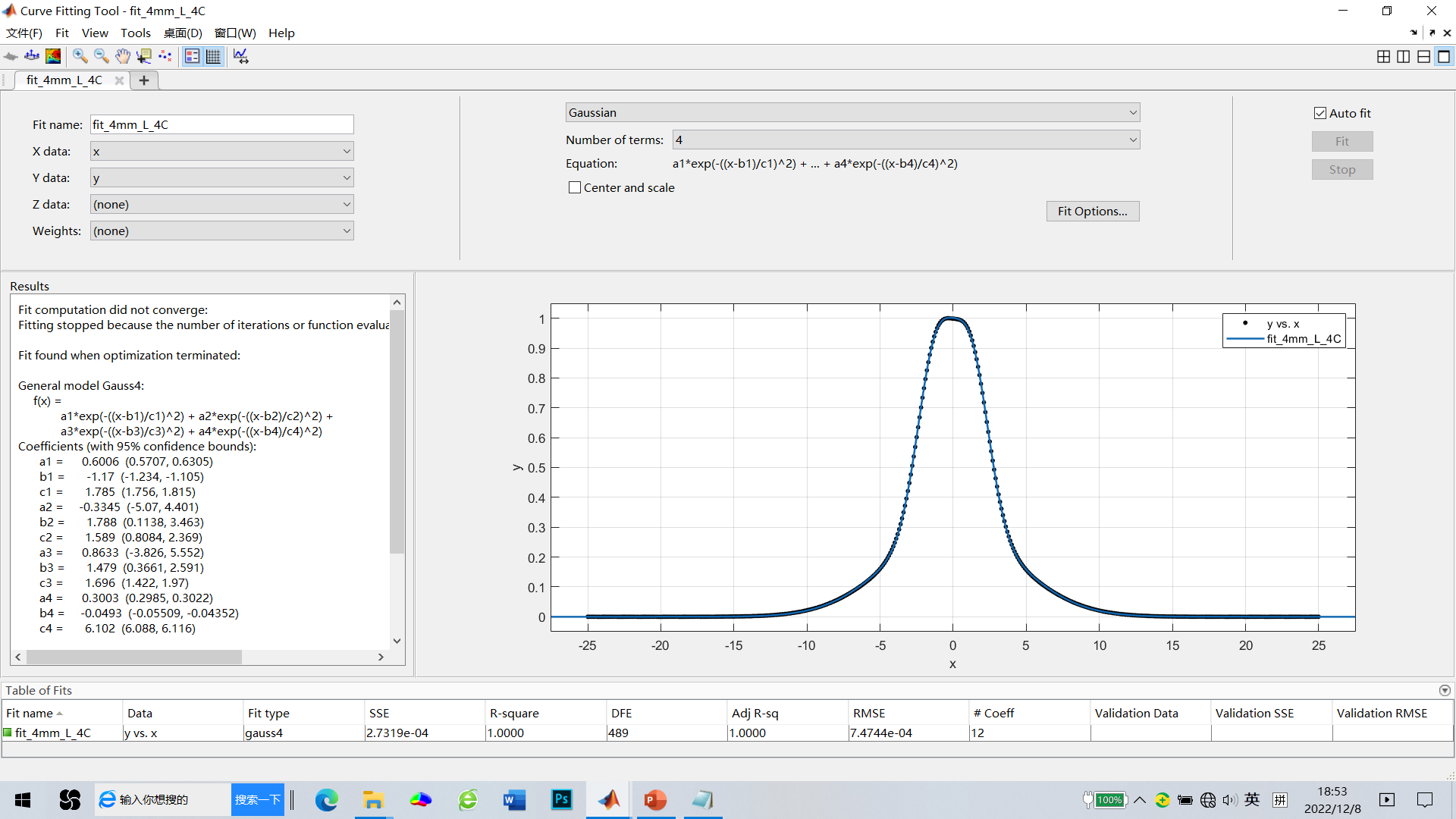


We resolved the dose profiles of the coefficient L for the 4 mm collimator to achieve the coefficients of the Gaussian function. With respect to $F_{L}\left( L \right)=\sum_{i=1}^{N} u_{i}e^{-\left( \frac{L-v_{i}}{w_{i}} \right)^{2}}$, here, a1-4 in MATLAB Curve Fitting Tool indicates *u_i_*. b1-4 indicates *v_i_*, and c1-4 indicates *w_i_*, N is equal to 4 for this case. Here, L can take negative values due to the purpose of fitting two-side dose falloff.

**Supplemental Figure A8**

**The relations between isodose (%) and L of the 4 mm collimator**


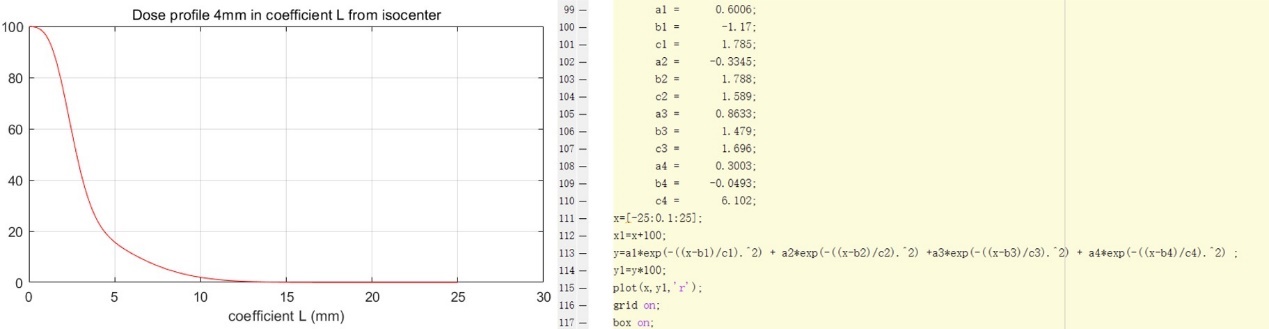


The relations between isodose (%) and coefficient L of the 4 mm collimator was calculated by the fitting coefficient. The left panel only shows the positive direction of dose falloff, when indicating the relationship between L and there-dimensional coordinate values in spatial distribution.

**Supplemental Figure A9**

**The coefficients of the 8 mm collimator in the x direction**


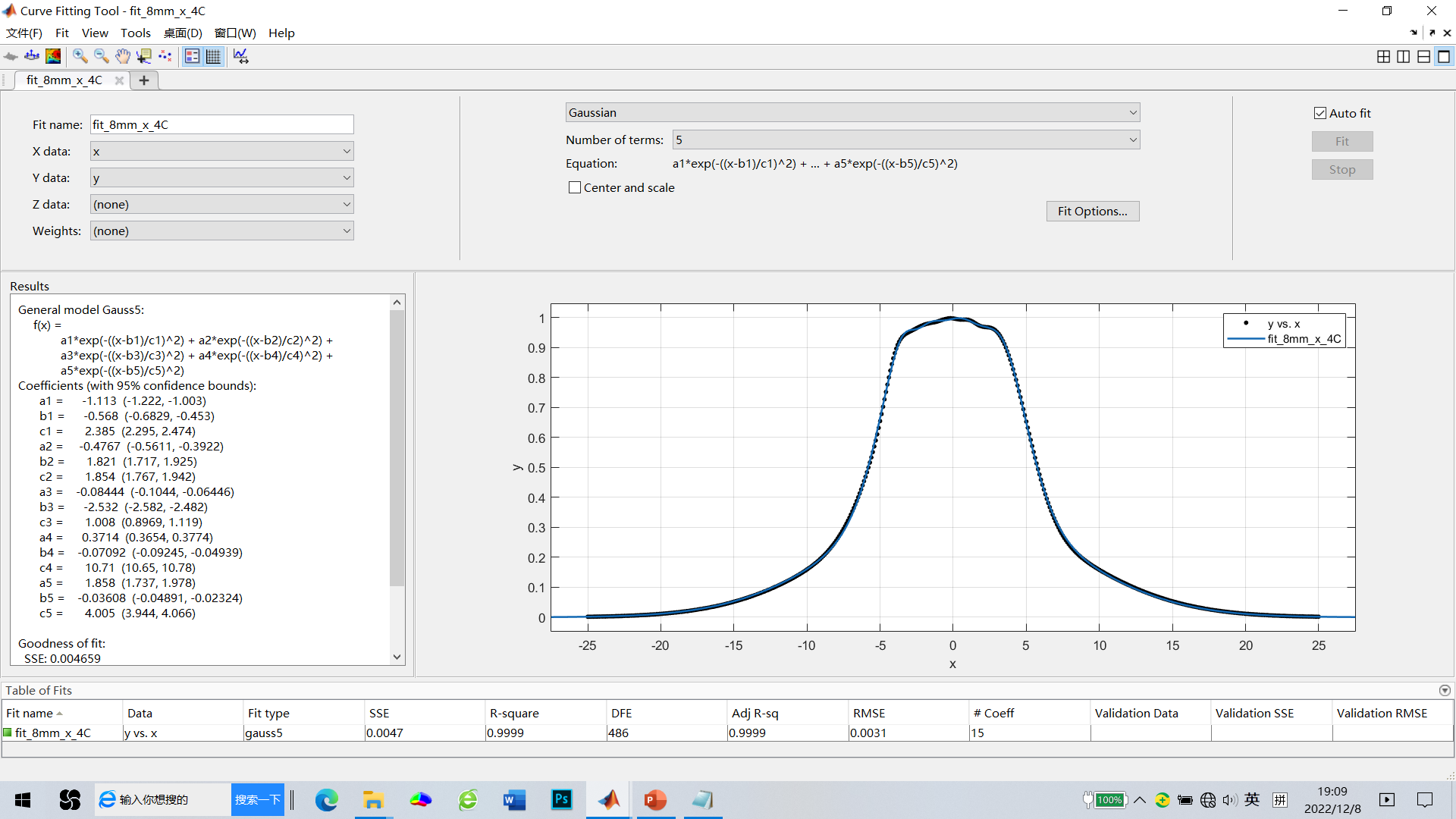


The curve in the dose profiles of the 8 mm collimator in the x direction was used to achieve the coefficients of the Gaussian function. With respect to $F_{x}\left( x_{a} \right)=\sum_{i=1}^{N} u_{i}e^{-\left( \frac{x_{a}-v_{i}}{w_{i}} \right)^{2}}$, here, a1-5 in MATLAB Curve Fitting Tool indicates *u_i_*. b1-5 indicates *v_i_*, and c1-5 indicates *w_i_*. N is equal to 5 for this case.

**Supplemental Figure A10**

**The relations between isodose (%) and x_a_ of the 8 mm collimator**


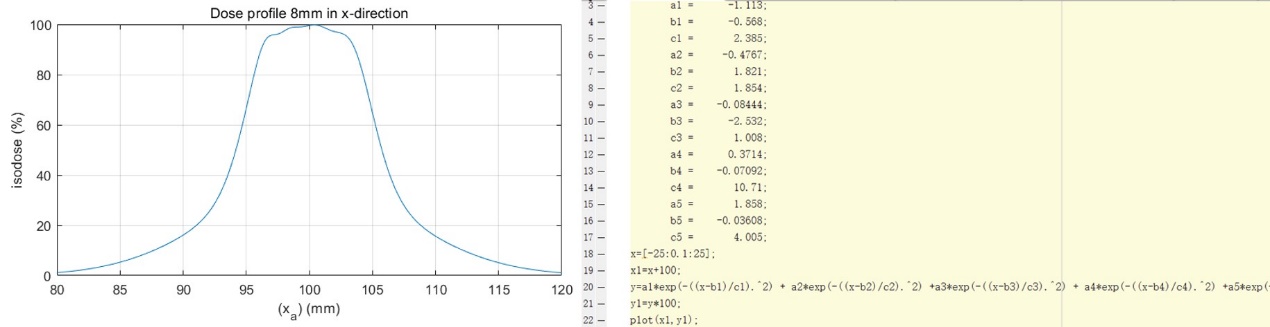


The relations between isodose (%) and x_a_ of the 8 mm collimator was calculated by the fitting coefficient.

**Supplemental Figure A11**

**The coefficients of the 8 mm collimator in the y direction**


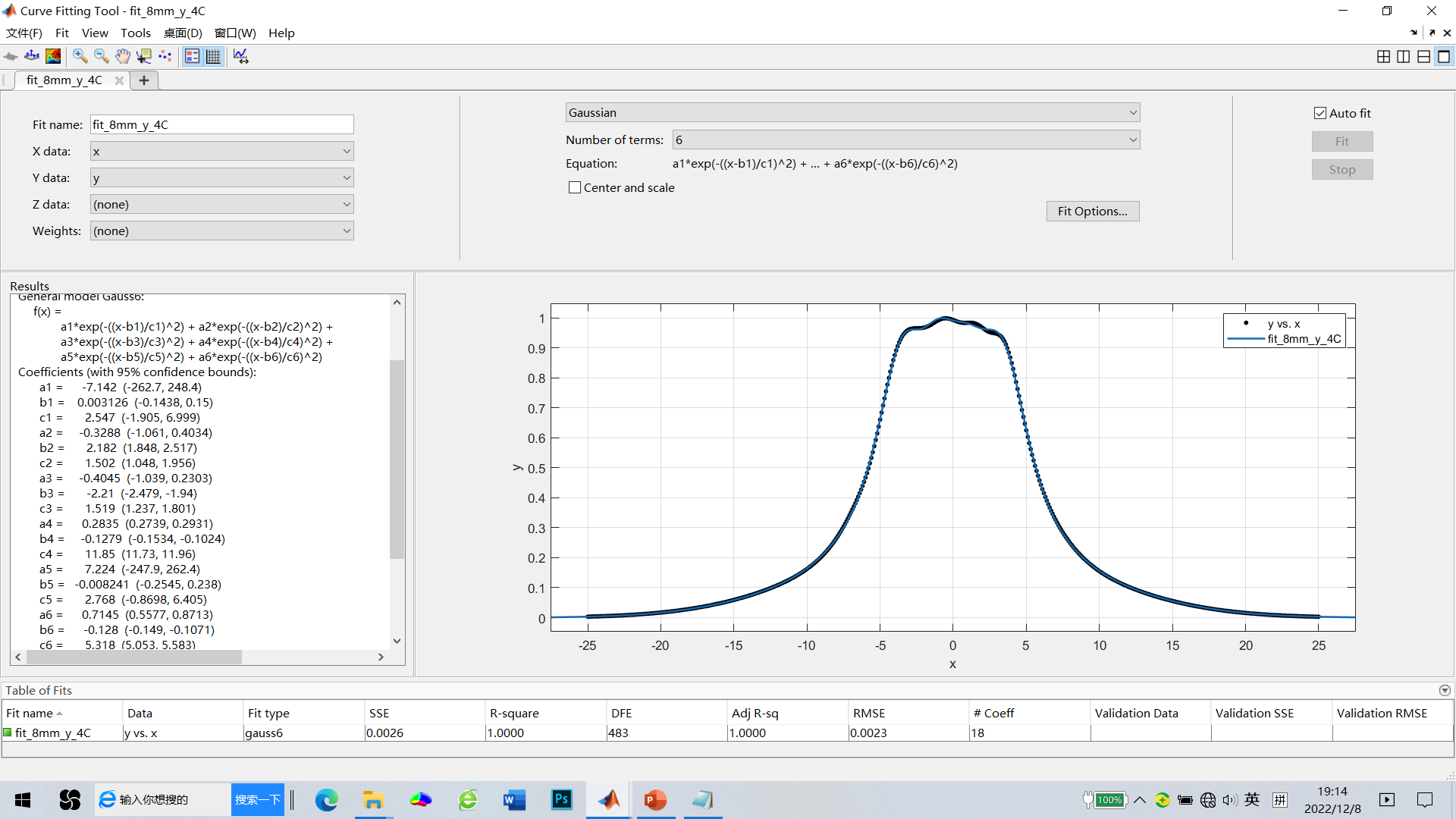


The curve in the dose profiles of the 8 mm collimator in the y direction was used to achieve the coefficients of the Gaussian function. With respect to $F_{y}\left( y_{b} \right)=\sum_{i=1}^{N} u_{i}e^{-\left( \frac{y_{b}-v_{i}}{w_{i}} \right)^{2}}$, here, a1-6 in MATLAB Curve Fitting Tool indicates *u_i_*. b1-6 indicates *v_i_*, and c1-6 indicates *w_i_*, N is equal to 6 for this case.

**Supplemental Figure A12**

**The relations between isodose (%) and y_b_ of the 8 mm collimator**


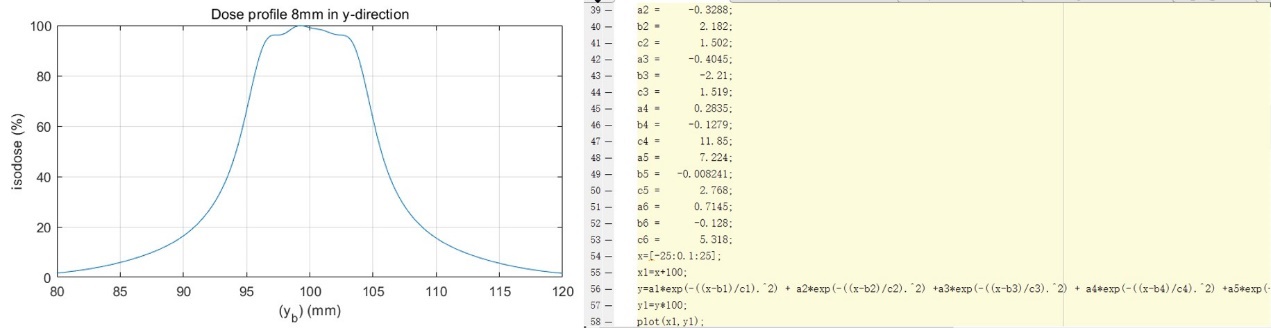


The relations between isodose (%) and y_b_ of the 8 mm collimator was calculated by the fitting coefficient.

**Supplemental Figure A13**

**The coefficients of the 8 mm collimator in the z direction**


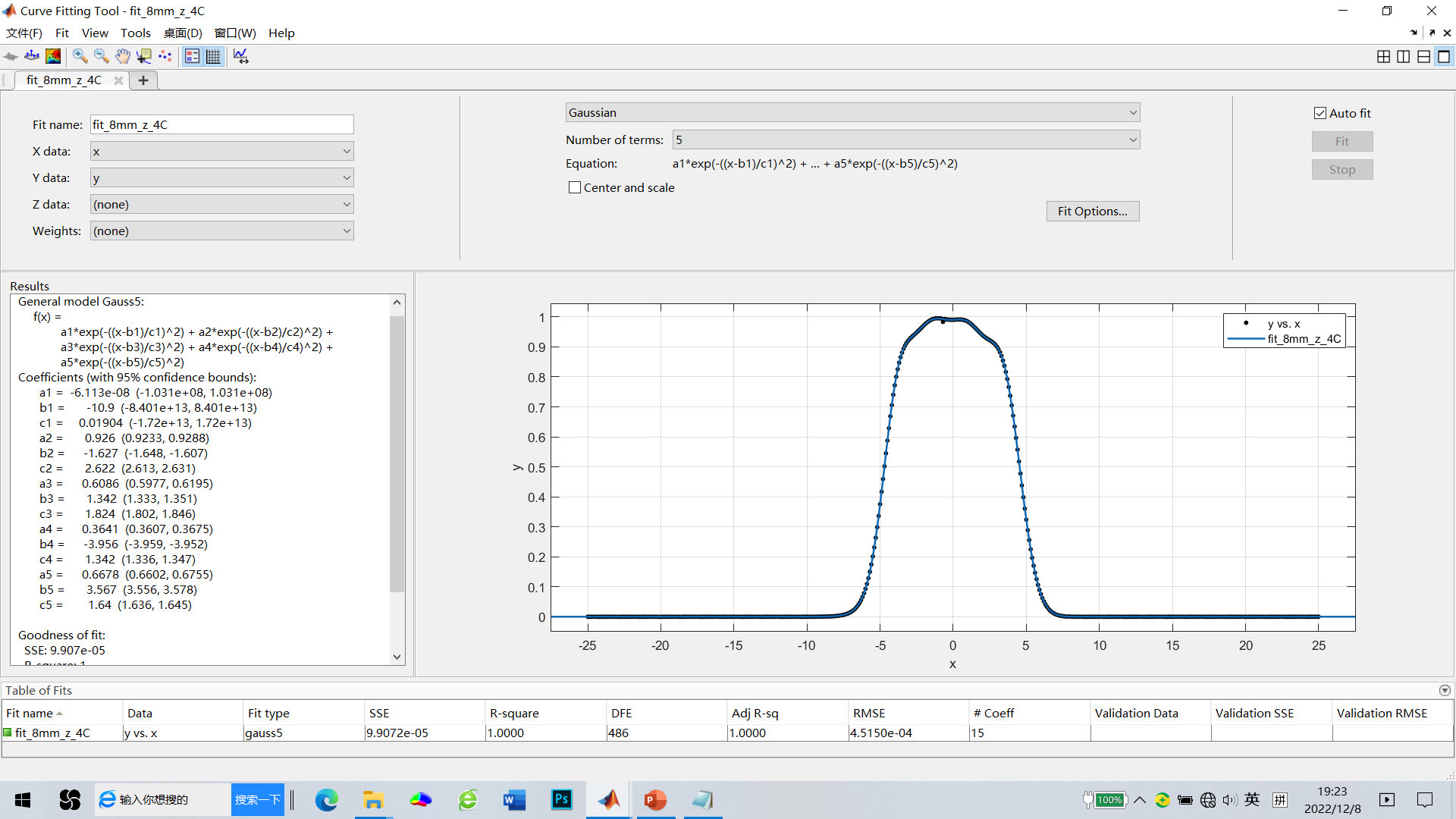


The curve in the dose profiles of the 8 mm collimator in the z direction was used to achieve the coefficients of the Gaussian function. With respect to $F_{z}\left( z_{c} \right)=\sum_{i=1}^{N} u_{i}e^{-\left( \frac{z_{c}-v_{i}}{w_{i}} \right)^{2}}$, here, a1-5 in MATLAB Curve Fitting Tool indicates *u_i_*. b1-5 indicates *v_i_*, and c1-6 indicates *w_i_*, N is equal to 5 for this case.

**Supplemental Figure A14**

**The relations between isodose (%) and z_c_ of the 8 mm collimator**


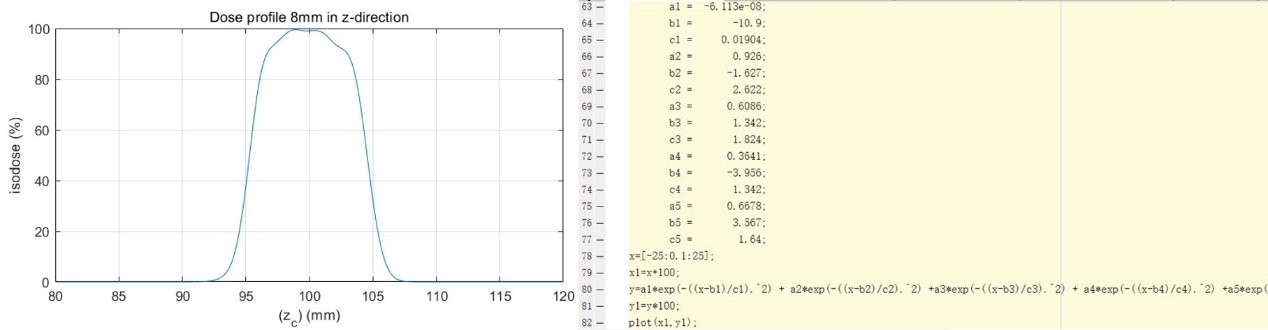


The relations between isodose (%) and z_c_ of the 8 mm collimator was calculated by the fitting coefficient.

**Supplemental Figure A15**

**The coefficients of the 8 mm collimator for L**


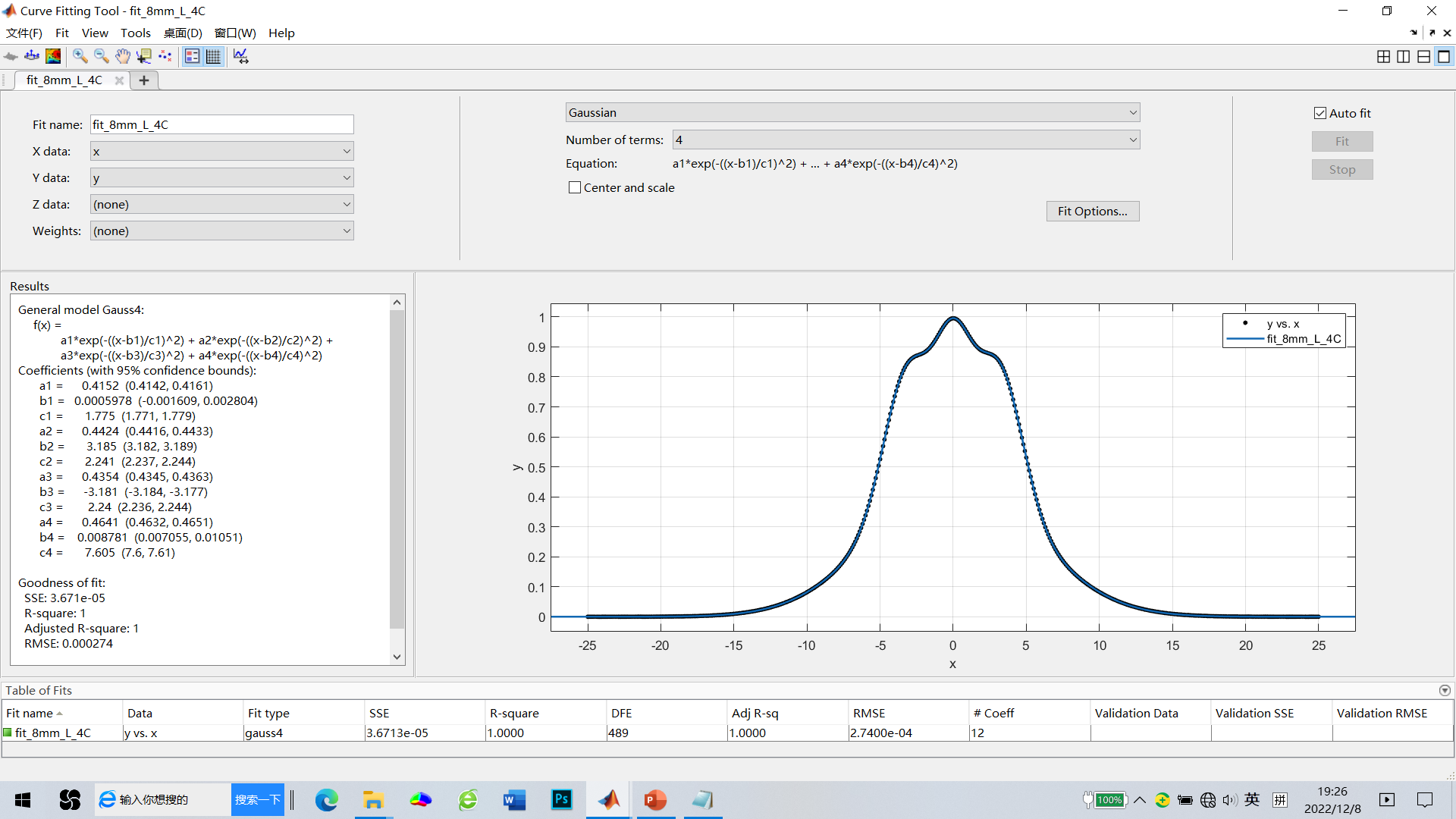


We resolved the dose profiles of the coefficient L for the 8 mm collimator to achieve the coefficients of the Gaussian function. With respect to $F_{L}\left( L \right)=\sum_{i=1}^{N} u_{i}e^{-\left( \frac{L-v_{i}}{w_{i}} \right)^{2}}$, here, a1-4 in MATLAB Curve Fitting Tool indicates *u_i_*. b1-4 indicates *v_i_*, and c1-4 indicates *w_i_*, N is equal to 4 for this case. Here, L can take negative values due to the purpose of fitting two-side dose falloff.

**Supplemental Figure A16**

**The relations between isodose (%) and L of the 8 mm collimator**


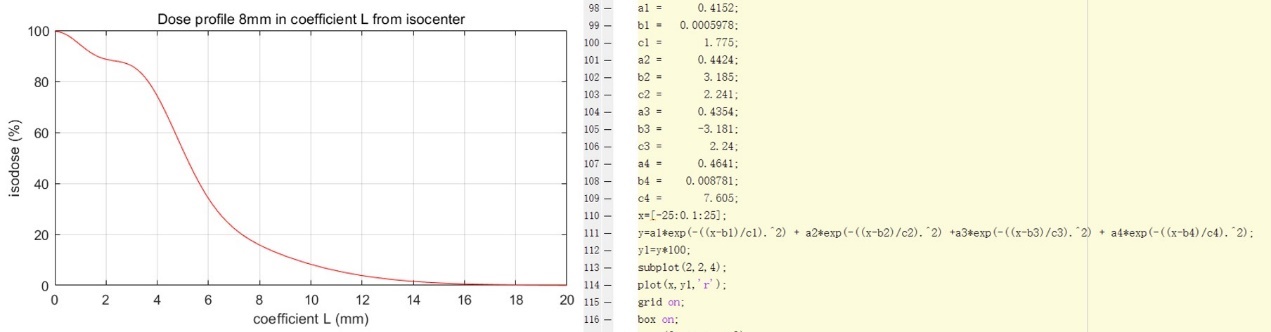


The relations between isodose (%) and coefficient L of the 8 mm collimator was calculated by the fitting coefficient. The left panel only shows the positive direction of dose falloff, when indicating the relationship between L and there-dimensional coordinate values in spatial distribution.

**Supplemental Figure A17**

**The coefficients of the 14 mm collimator in the x direction**


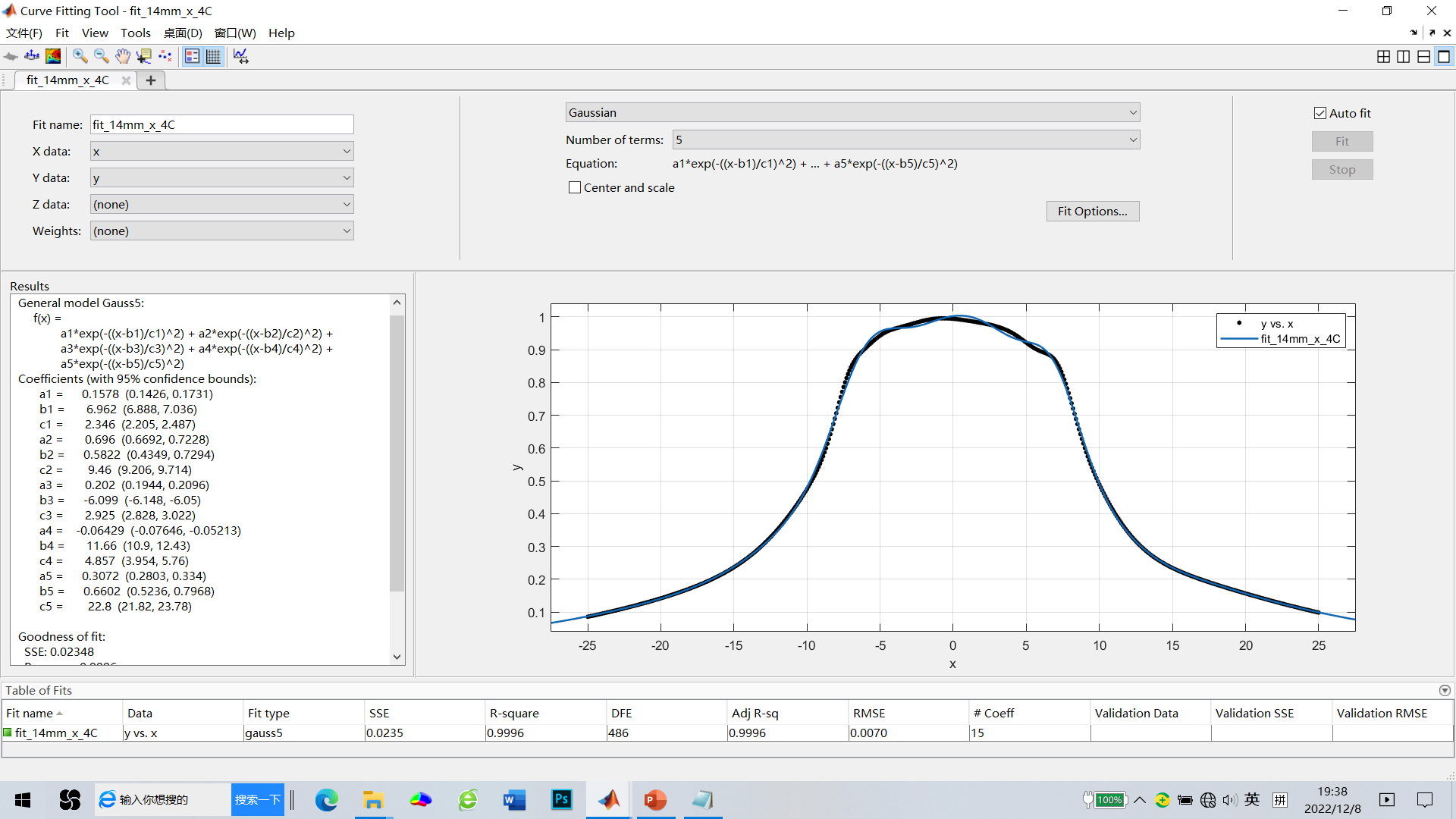


The curve in the dose profiles of the 14 mm collimator in the x direction was used to achieve the coefficients of the Gaussian function. With respect to $F_{x}\left( x_{a} \right)=\sum_{i=1}^{N} u_{i}e^{-\left( \frac{x_{a}-v_{i}}{w_{i}} \right)^{2}}$, here, a1-5 in MATLAB Curve Fitting Tool indicates *u_i_*. b1-5 indicates *v_i_*, and c1-5 indicates *w_i_*. N is equal to 5 for this case.

**Supplemental Figure A18**

**The relations between isodose (%) and x_a_ of the 14 mm collimator**


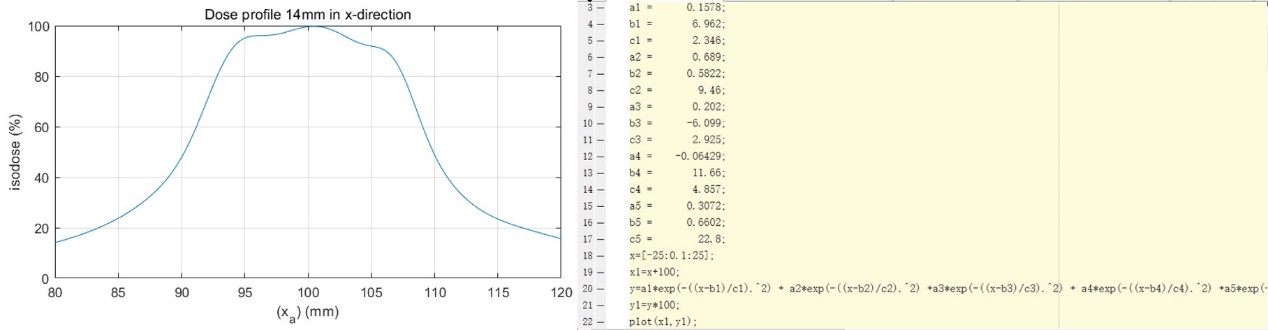


The relations between isodose (%) and x_a_ of the 14 mm collimator was calculated by the fitting coefficient.

**Supplemental Figure A19**

**The coefficients of the 14 mm collimator in the y direction**


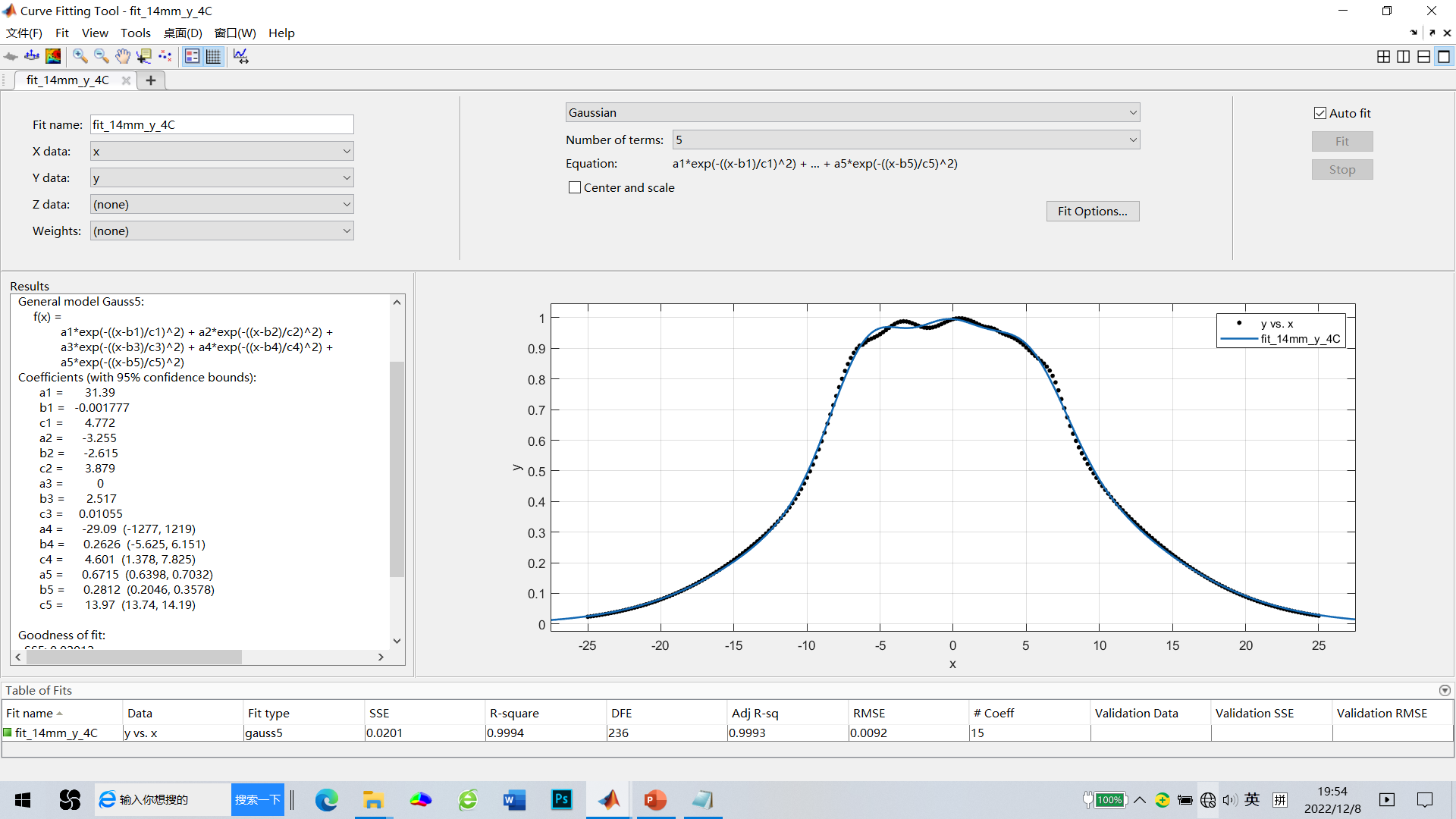


The curve in the dose profiles of the 14 mm collimator in the y direction was used to achieve the coefficients of the Gaussian function. With respect to $F_{y}\left( y_{b} \right)=\sum_{i=1}^{N} u_{i}e^{-\left( \frac{y_{b}-v_{i}}{w_{i}} \right)^{2}}$, here, a1-5 in MATLAB Curve Fitting Tool indicates *u_i_*. b1-5 indicates *v_i_*, and c1-5 indicates *w_i_*, N is equal to 5 for this case.

**Supplemental Figure A20**

**The relations between isodose (%) and y_b_ of the 14 mm collimator**


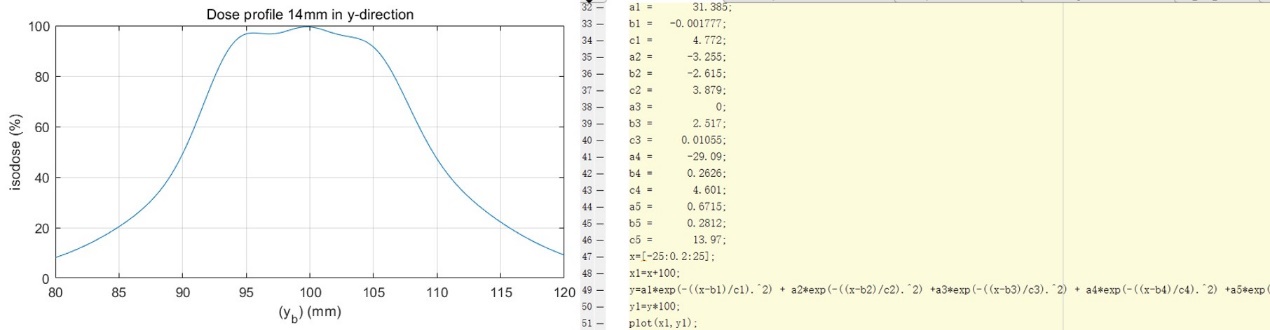


The relations between isodose (%) and y_b_ of the 14 mm collimator was calculated by the fitting coefficient.

**Supplemental Figure A21**

**The coefficients of the 14 mm collimator in the z direction**


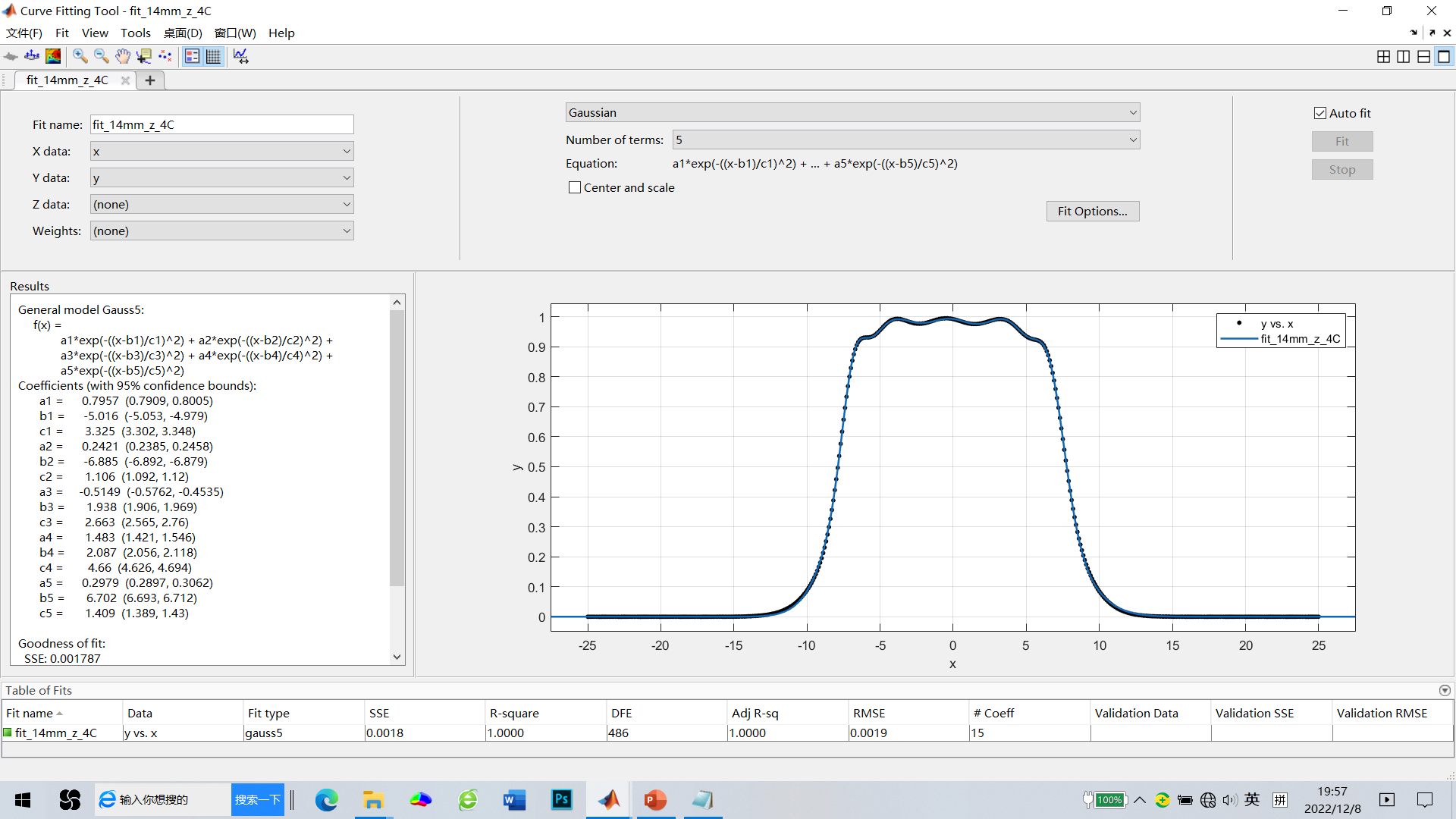


The curve in the dose profiles of the 14 mm collimator in the z direction was used to achieve the coefficients of the Gaussian function. With respect to $F_{z}\left( z_{c} \right)=\sum_{i=1}^{N} u_{i}e^{-\left( \frac{z_{c}-v_{i}}{w_{i}} \right)^{2}}$, here, a1-5 in MATLAB Curve Fitting Tool indicates *u_i_*. b1-5 indicates *v_i_*, and c1-6 indicates *w_i_*, N is equal to 5 for this case.

**Supplemental Figure A22**

**The relations between isodose (%) and z_c_ of the 14 mm collimator**


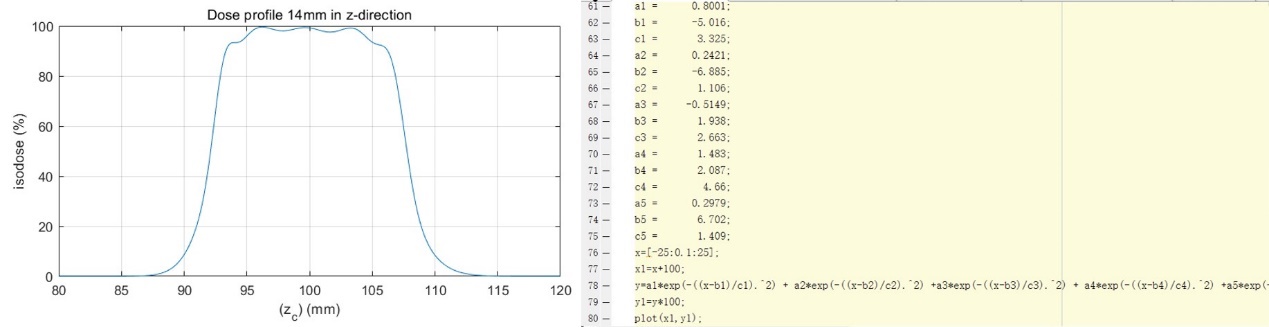


The relations between isodose (%) and z_c_ of the 14 mm collimator was calculated by the fitting coefficient.

**Supplemental Figure A23**

**The coefficients of the 14 mm collimator for L**


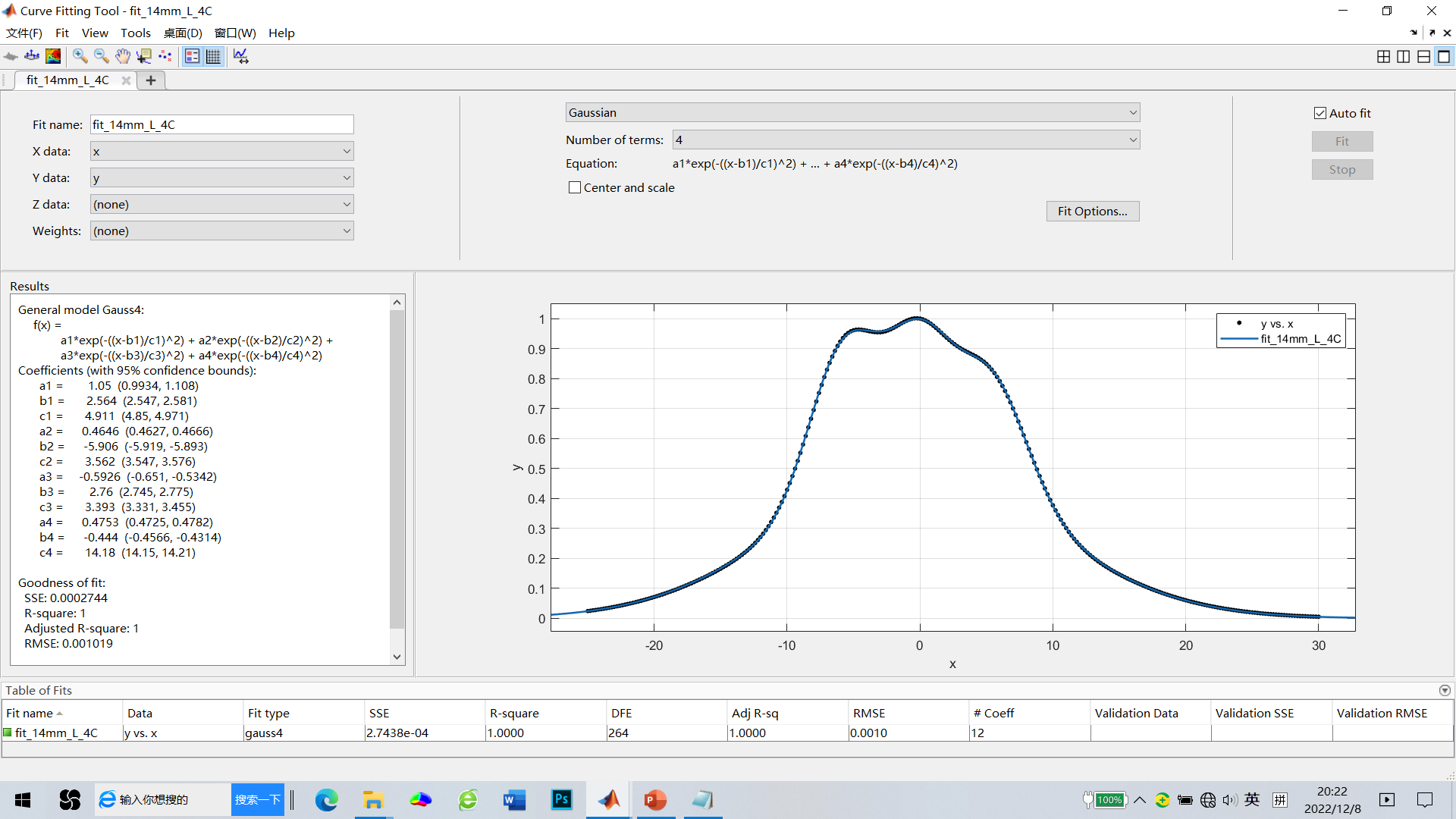


We resolved the dose profiles of the coefficient L for the 14 mm collimator to achieve the coefficients of the Gaussian function. With respect to $F_{L}\left( L \right)=\sum_{i=1}^{N} u_{i}e^{-\left( \frac{L-v_{i}}{w_{i}} \right)^{2}}$, here, a1-4 in MATLAB Curve Fitting Tool indicates *u_i_*. b1-4 indicates *v_i_*, and c1-4 indicates *w_i_*, N is equal to 4 for this case. Here, L can take negative values due to the purpose of fitting two-side dose falloff.

**Supplemental Figure A24**

**The relations between isodose (%) and L of the 14 mm collimator**


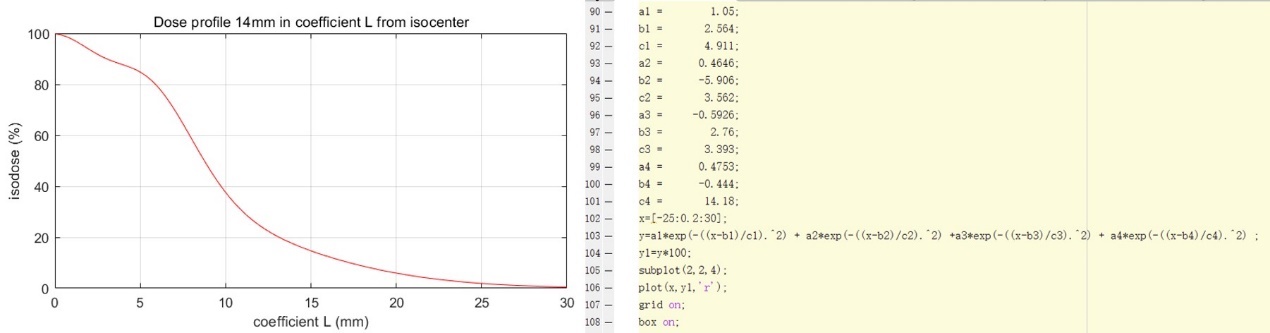


The relations between isodose (%) and coefficient L of the 14 mm collimator was calculated by the fitting coefficient. The left panel only shows the positive direction of dose falloff, when indicating the relationship between L and there-dimensional coordinate values in spatial distribution.

**Supplemental Figure A25**

**The coefficients of the 18 mm collimator in the x direction**


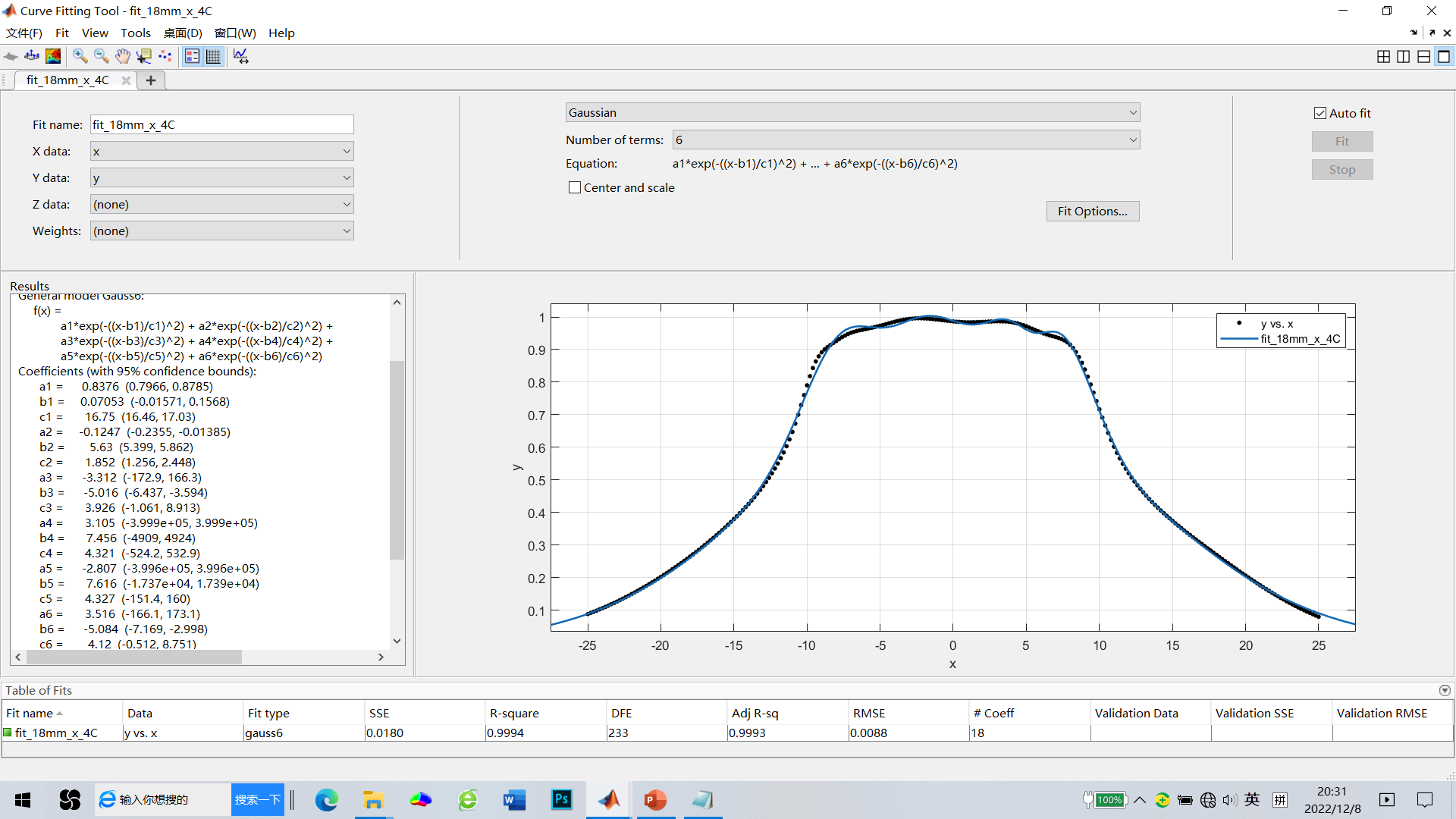


The curve in the dose profiles of the 18 mm collimator in the x direction was used to achieve the coefficients of the Gaussian function. With respect to $F_{x}\left( x_{a} \right)=\sum_{i=1}^{N} u_{i}e^{-\left( \frac{x_{a}-v_{i}}{w_{i}} \right)^{2}}$, here, a1-6 in MATLAB Curve Fitting Tool indicates *u_i_*. b1-6 indicates *v_i_*, and c1-6 indicates *w_i_*. N is equal to 6 for this case.

**Supplemental Figure A26**

**The relations between isodose (%) and x_a_ of the 18 mm collimator**


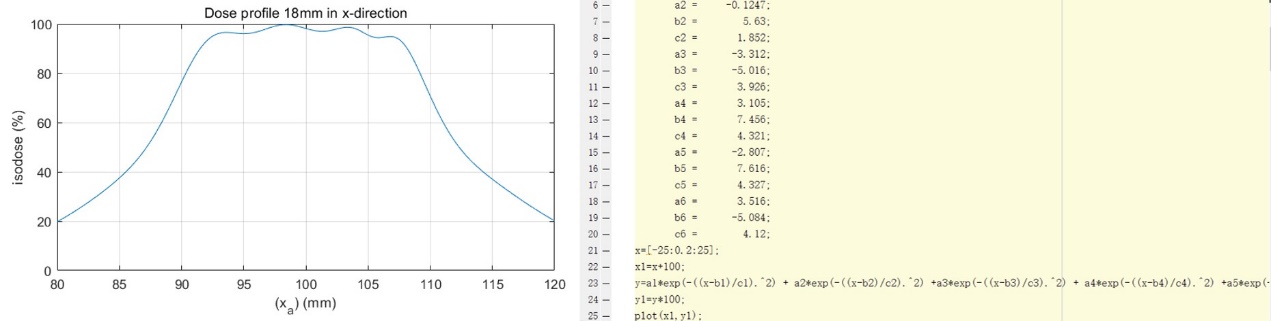


The relations between isodose (%) and x_a_ of the 18 mm collimator was calculated by the fitting coefficient.

**Supplemental Figure A27**

**The coefficients of the 18 mm collimator in the y direction**


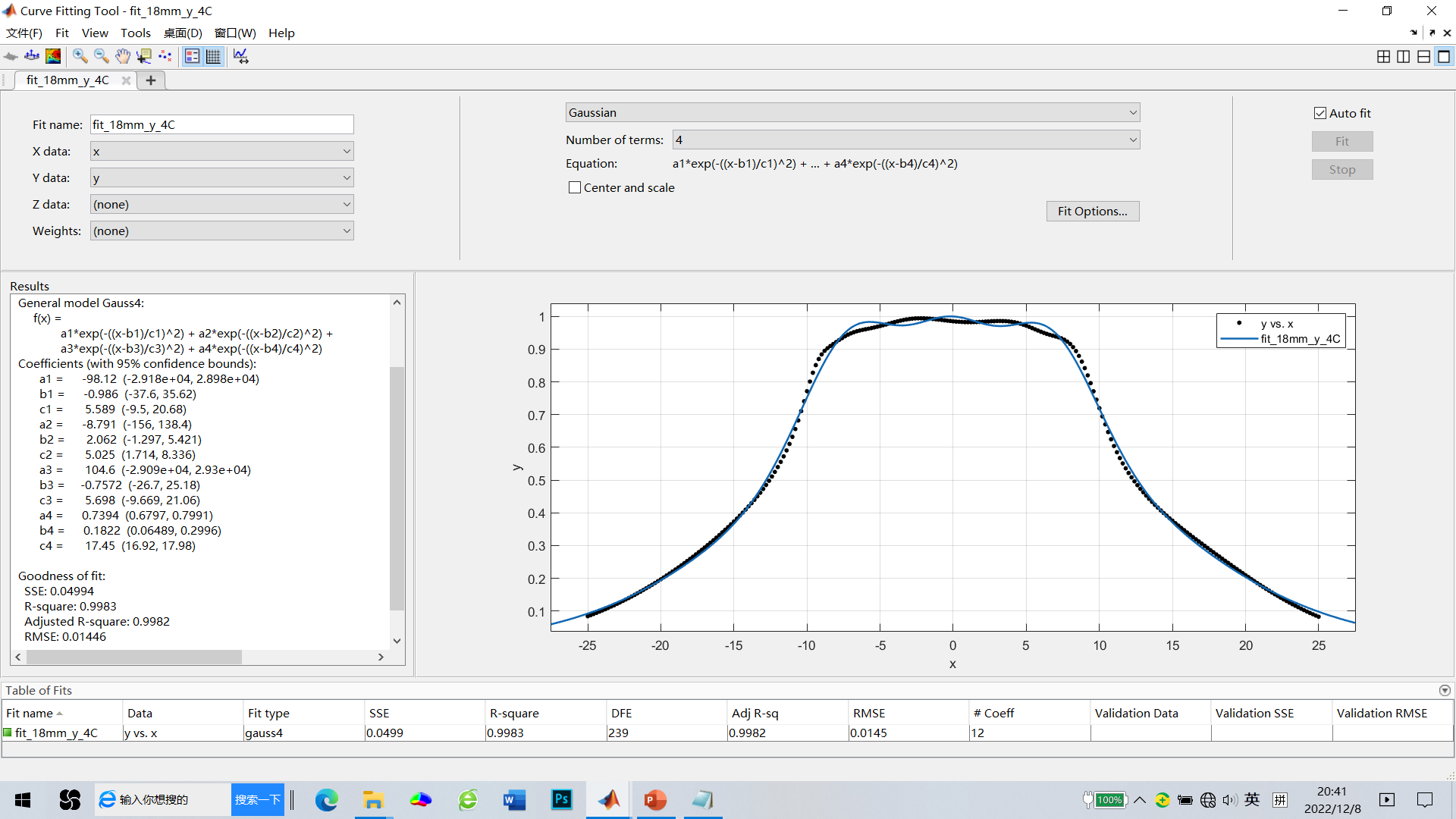


The curve in the dose profiles of the 18 mm collimator in the y direction was used to achieve the coefficients of the Gaussian function. With respect to $F_{y}\left( y_{b} \right)=\sum_{i=1}^{N} u_{i}e^{-\left( \frac{y_{b}-v_{i}}{w_{i}} \right)^{2}}$, here, a1-4 in MATLAB Curve Fitting Tool indicates *u_i_*. b1-4 indicates *v_i_*, and c1-4 indicates *w_i_*, N is equal to 4 for this case.

**Supplemental Figure A28**

**The relations between isodose (%) and y_b_ of the 18 mm collimator**


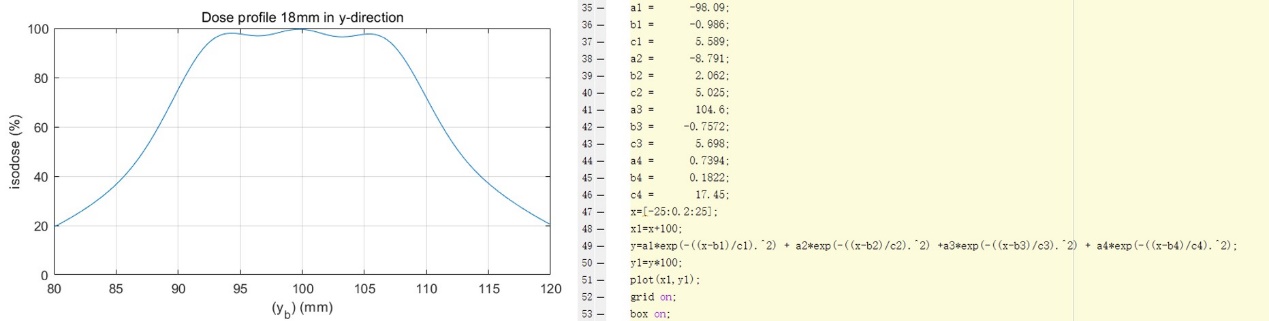


The relations between isodose (%) and y_b_ of the 18 mm collimator was calculated by the fitting coefficient.

**Supplemental Figure A29**

**The coefficients of the 18 mm collimator in the z direction**


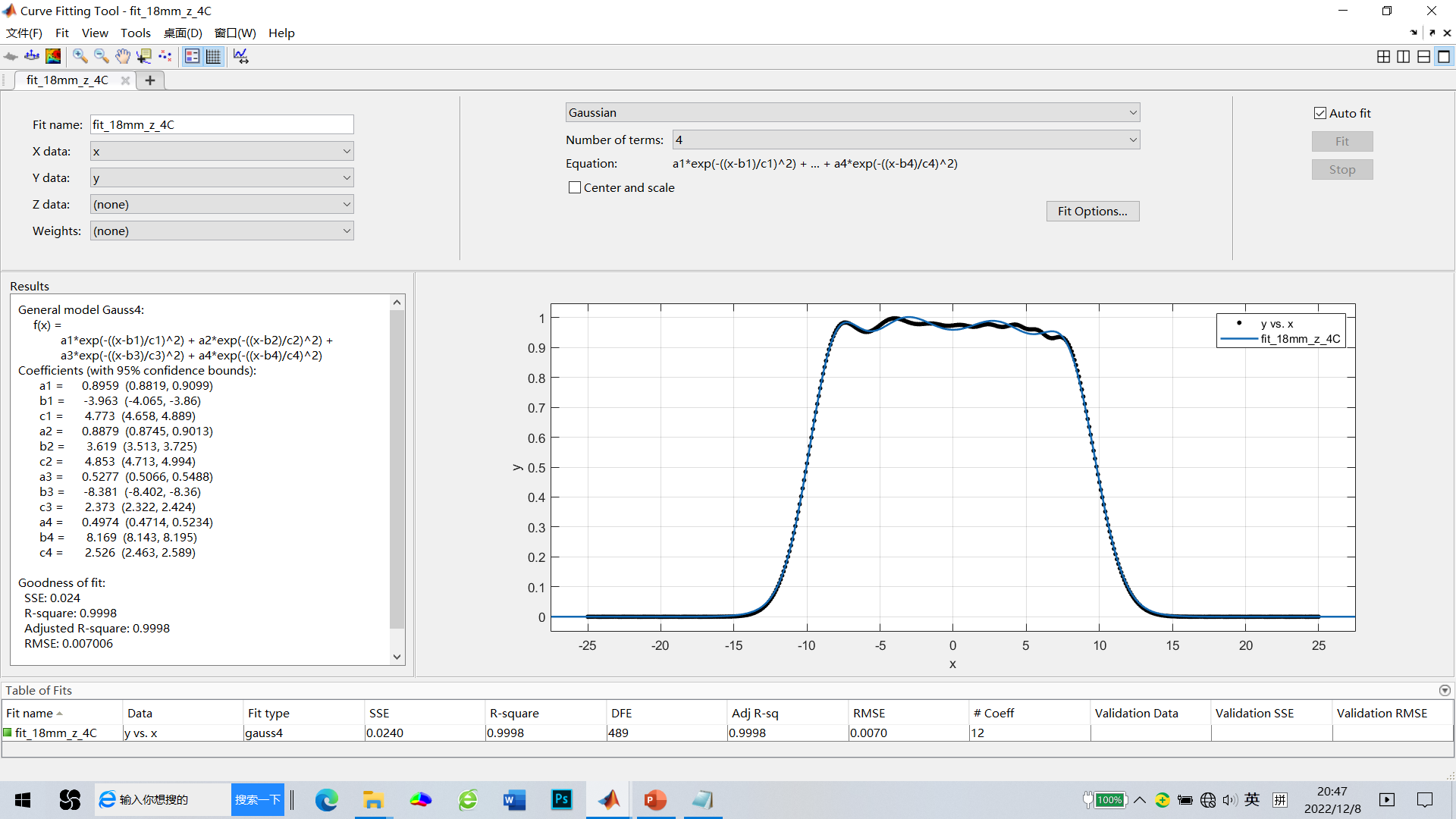


The curve in the dose profiles of the 18 mm collimator in the z direction was used to achieve the coefficients of the Gaussian function. With respect to $F_{z}\left( z_{c} \right)=\sum_{i=1}^{N} u_{i}e^{-\left( \frac{z_{c}-v_{i}}{w_{i}} \right)^{2}}$, here, a1-4 in MATLAB Curve Fitting Tool indicates *u_i_*. b1-4 indicates *v_i_*, and c1-4 indicates *w_i_*, N is equal to 4 for this case.

**Supplemental Figure A30**

**The relations between isodose (%) and z_c_ of the 18 mm collimator**


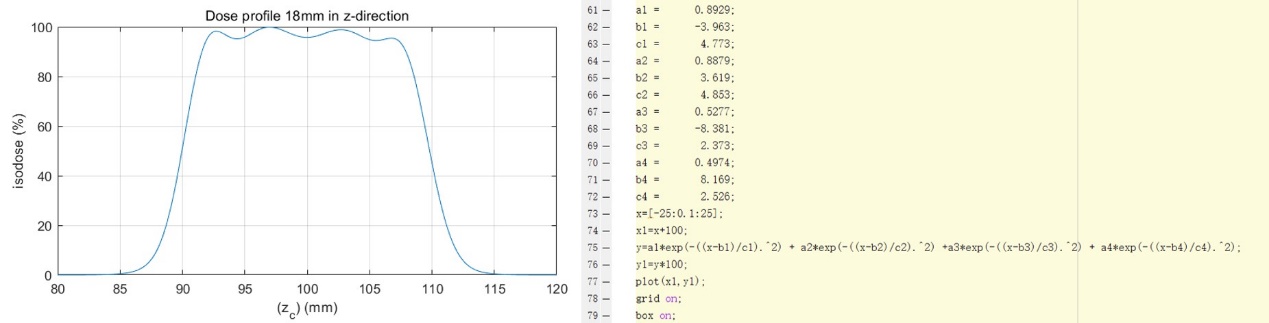


The relations between isodose (%) and z_c_ of the 18 mm collimator was calculated by the fitting coefficient.

**Supplemental Figure A31**

**The coefficients of the 18 mm collimator for L**


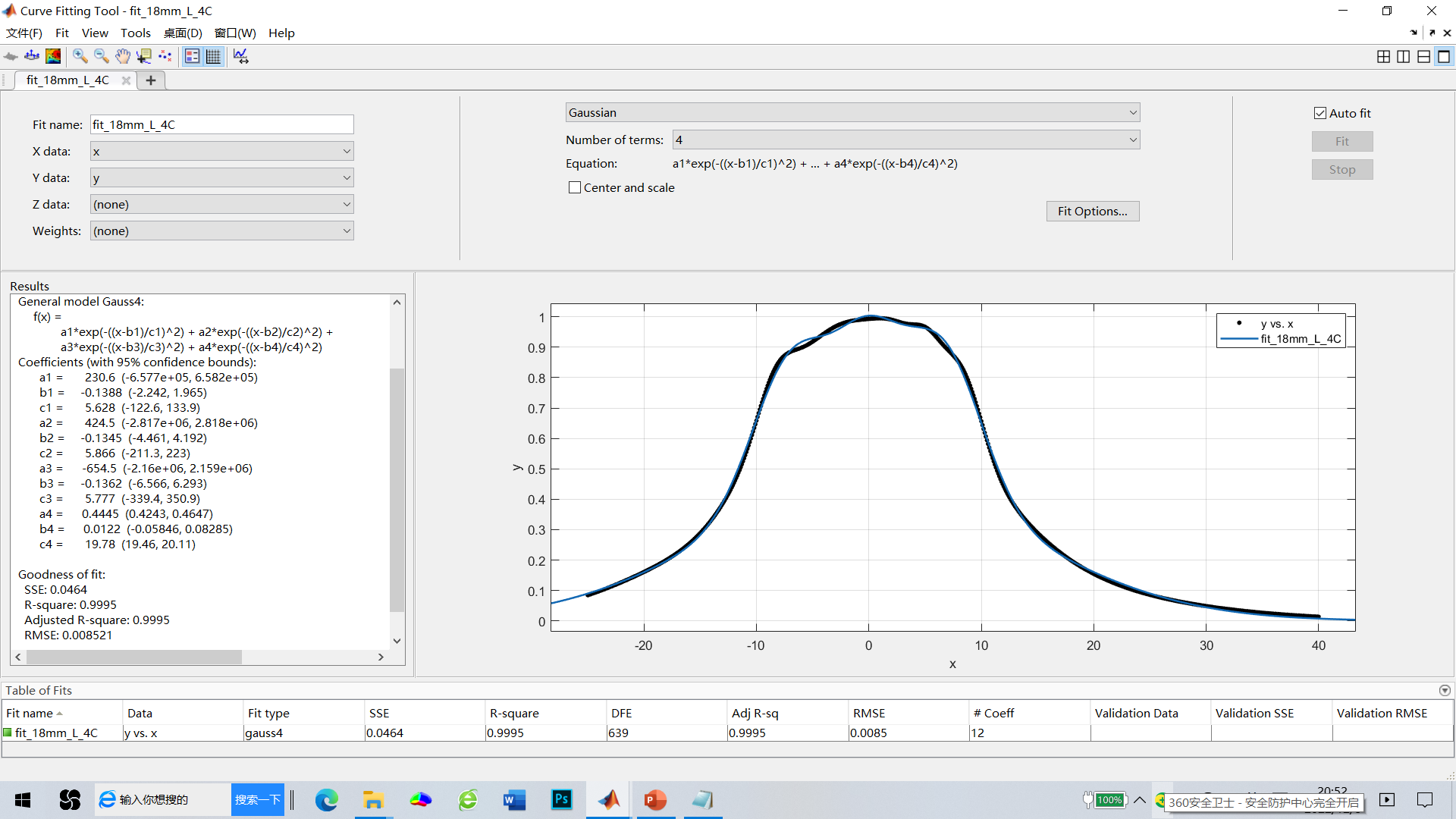


We resolved the dose profiles of the coefficient L for the 18 mm collimator to achieve the coefficients of the Gaussian function. With respect to $F_{L}\left( L \right)=\sum_{i=1}^{N} u_{i}e^{-\left( \frac{L-v_{i}}{w_{i}} \right)^{2}}$, here, a1-4 in MATLAB Curve Fitting Tool indicates *u_i_*. b1-4 indicates *v_i_*, and c1-4 indicates *w_i_*, N is equal to 4 for this case. Here, L can take negative values due to the purpose of fitting two-side dose falloff.

**Supplemental Figure A32**

**The relations between isodose (%) and L of the 18 mm collimator**


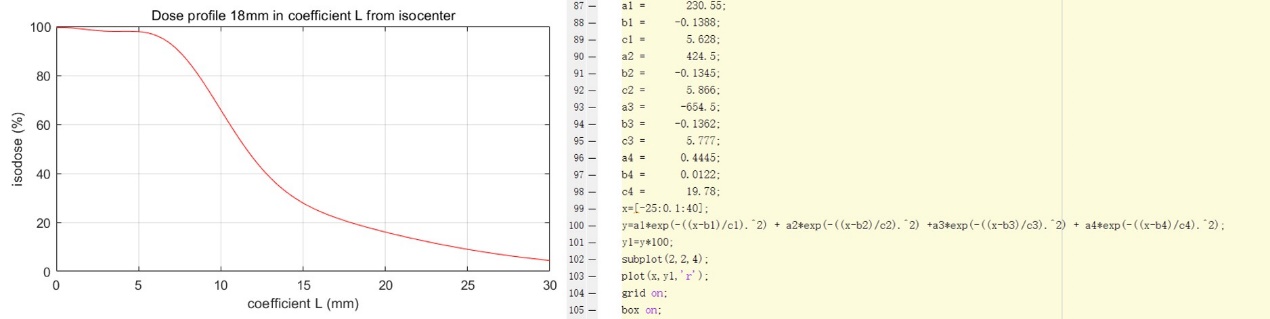


The relations between isodose (%) and coefficient L of the 18 mm collimator was calculated by the fitting coefficient. The left panel only shows the positive direction of dose falloff, when indicating the relationship between L and there-dimensional coordinate values in spatial distribution.

**References**

[1] Elekta. A new TMR algorithm in Leksell GammaPlan [white paper]. 2011.

[2] Osmancikova P, Novotny J, Jr., Solc J, Pipek J. Comparison of the Convolution algorithm with TMR10 for Leksell Gamma knife and dosimetric verification with radiochromic gel dosimeter. J Appl Clin Med Phys. 2018;19:138-44. <https://doi.org/10.1002/acm2.12238>

[3] Chung HT, Park JH, Chun KJ. Verification of dose profiles generated by the convolution algorithm of the gamma knife((R)) radiosurgery planning system. Med Phys. 2017;44:4880-9. <https://doi.org/10.1002/mp.12347>
