# Supplementary material for: MatBED_B&C: A 3-dimensional biologically effective dose analytic approach for the retrospective study of gamma knife radiosurgery in a B&C model
Source: MethodsX. 2023 Aug 5;11:102320. doi: 10.1016/j.mex.2023.102320 (PMC10433126; doi:10.1016/j.mex.2023.102320)

***Supplementary Material B***

**The workflow of 3-dimensional biologically effective dose analysis**

According to the dose profiles of an iso-centre, we visualized a shot for the 4 mm, 8 mm, 14 mm, and 18 mm collimator of the B&C model.

**Supplemental Figure B1**

**The shot contour for 4 mm collimator**


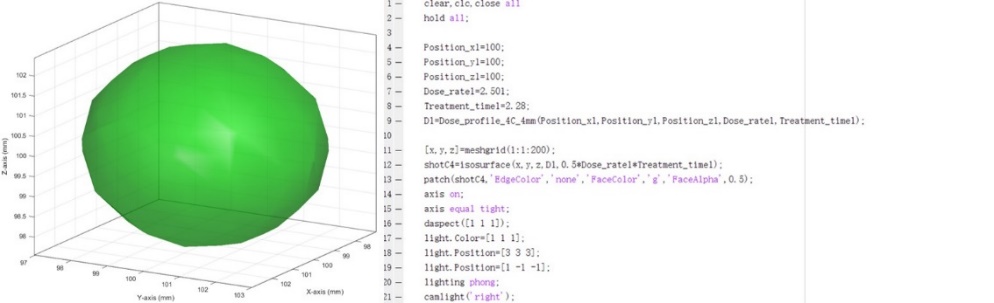


**Supplemental Figure B2**

**The shot contour for 8 mm collimator**


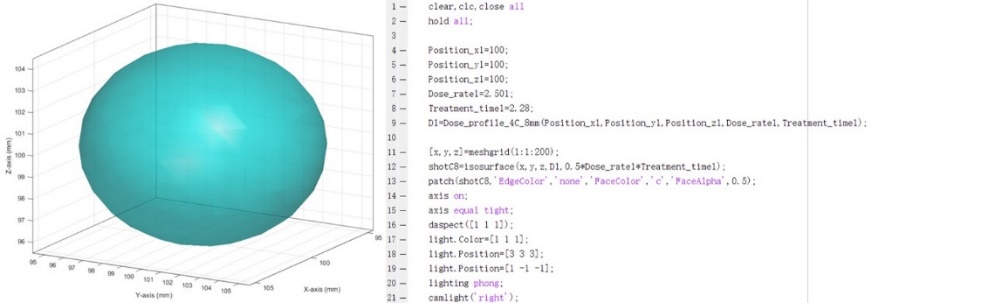


**Supplemental Figure B3**

**The shot contour for 14 mm collimator**


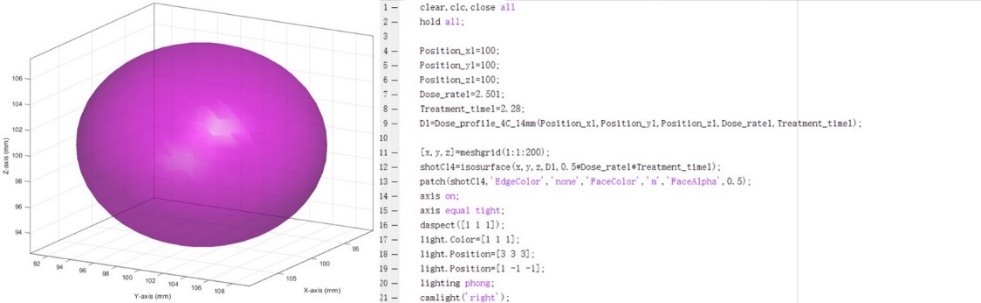


**Supplemental Figure B4**

**The shot contour for 18 mm collimator**


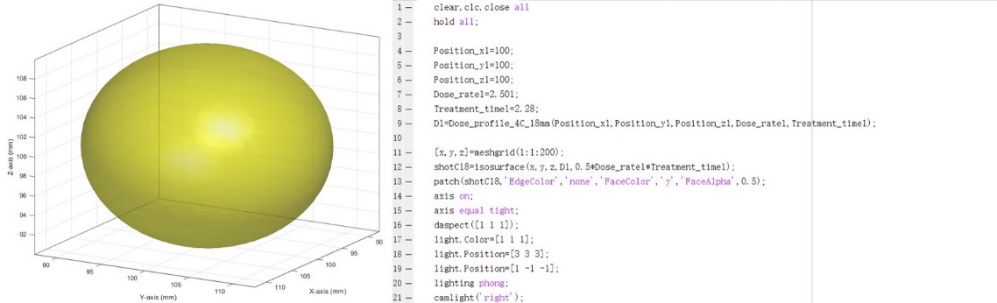


The spatial morphology and position of a shot could be visualized using the 3-dimensional coordinate values of each iso-centre. The iso-dose surface visualized the total dose of all shots.

**Supplemental Figure B5**

**Shot contours for 8 mm and 18 mm collimators**


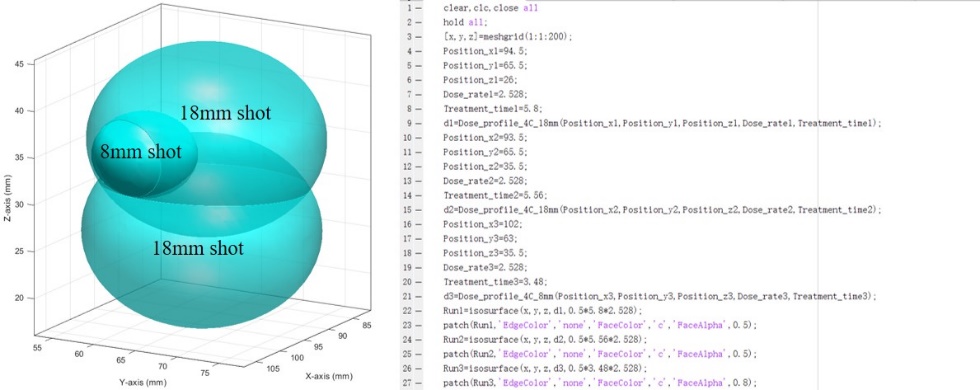


The example shows that the shots are visualized using the 3-dimensional coordinate values of each iso-centre of the B&C model.

**Supplemental Figure B6**

**The contour of the prescription iso-dose surface**


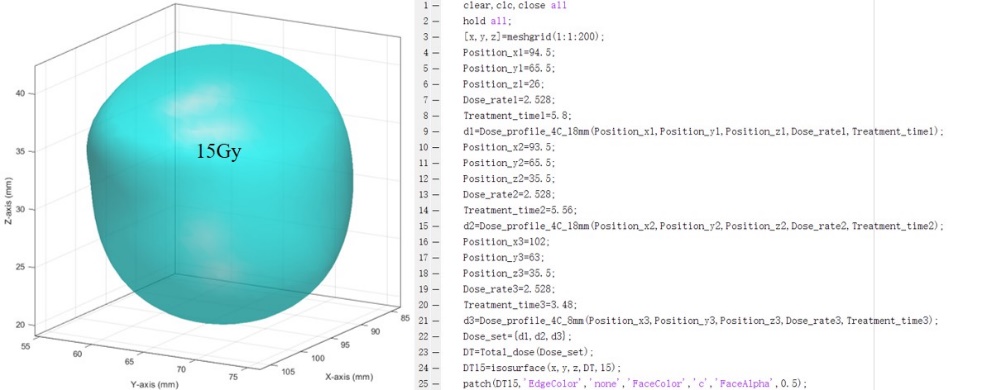


The example shows 15 Gy at 40% iso-surface.

Accordingly, we visualized the spatial distribution of biologically effective dose (BED)

**Supplemental Figure B7**

**Visualization of 3-dimensional BED**


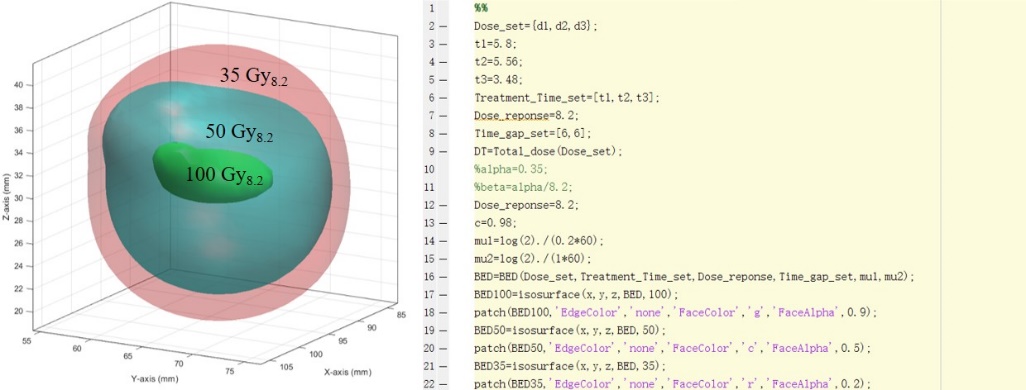


The example shows iso-surfaces of 35 Gy_8.2_ (red contour), 50 Gy_8.2_ (cyan contour), and 100 Gy_8.2_ (green contour)

If we inputted a 3D model of a radiosurgical target, we could generate the BED-volume histograms according to the 3D BED distributions.

**Supplemental Figure B8**

**Formation of differential BED-volume histograms**


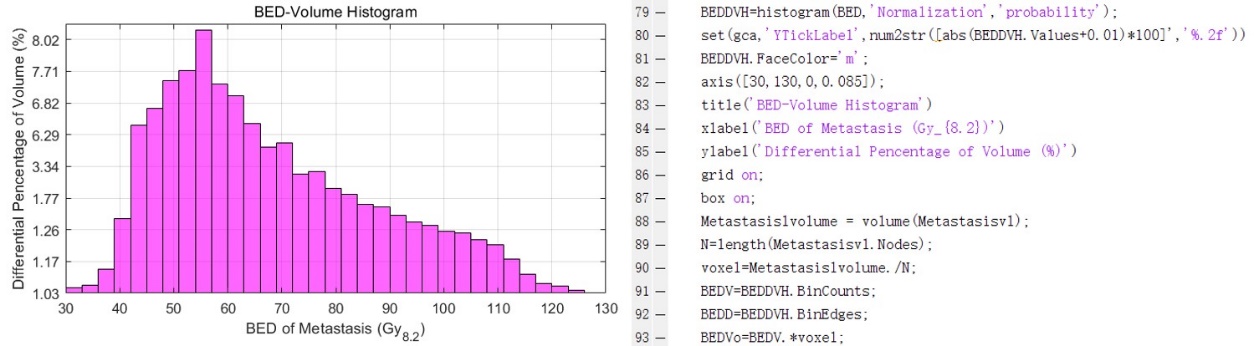


**Supplemental Figure B9**

**Formation of cumulative BED-volume histograms**


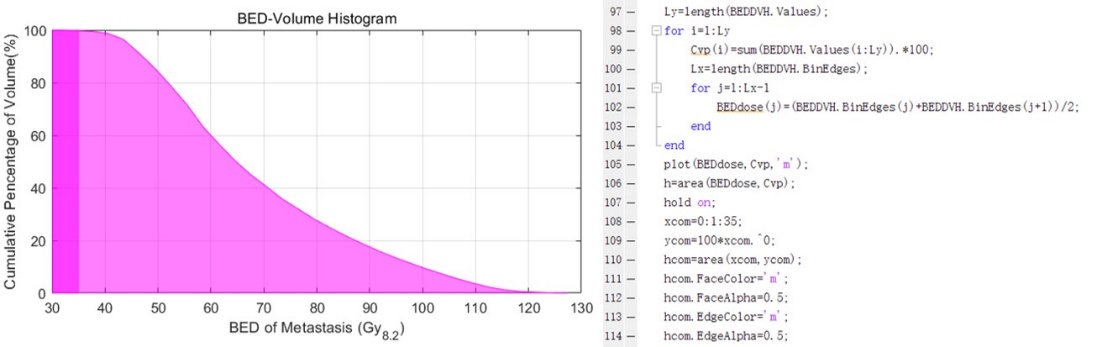

Supplement: Supplementary file 2 [file mmc2.docx]
